# KIAA1522 potentiates TNFα-NFκB signaling to antagonize platinum-based chemotherapy in lung adenocarcinoma

**DOI:** 10.1186/s13046-020-01684-x

**Published:** 2020-08-27

**Authors:** Boshi Wang, Tiantian Jing, Weilin Jin, Jinnan Chen, Chengsi Wu, Mingrong Wang, Yizhen Liu

**Affiliations:** 1 State Key Laboratory of Oncogenes and Related Genes, Shanghai Cancer Institute, Renji Hospital, Shanghai Jiao Tong University School of Medicine, Shanghai, 200032 China; 2 Institute of Nano Biomedicine and Engineering, Department of Instrument Science and Engineering, Key Laboratory for Thin Film and Microfabrication Technology of Ministry of Education, School of Electronic Information and Electronic Engineering, Shanghai Jiaotong University, Shanghai, 200240 China; 3 Renji Hospital, School of Medicine, Shanghai Jiao Tong University, Shanghai Institute of Digestive Disease, Shanghai, China; 4 State Key Laboratory of Molecular Oncology, Center for Cancer Precision Medicine, National Cancer Center/National Clinical Research Center for Cancer/Cancer Hospital, Chinese Academy of Medical Sciences, Peking Union Medical College, Beijing, 100021 China; 5 Department of Medical Oncology, Fudan University Shanghai Cancer Center, Shanghai, 200032 China; 6 Department of Oncology, Shanghai Medical College, Fudan University, Shanghai, 200032 China

**Keywords:** KIAA1522, Lung adenocarcinoma, NFκB, Chemoresistance, Platinum-based chemotherapy

## Abstract

**Background:**

The platinum-based chemotherapy is the first-line regimen for the treatment of Non-small cell lung cancer (NSCLC). However, the therapeutic efficiency is largely limited by tenacious chemo-insensitivity that results in inferior prognosis in a cohort of patients. It has been known that KIAA1522 is aberrantly expressed and implicated in several types of solid tumors including NSCLC. Nowadays, knowledge about this gene is quite limited. Here, we aimed to identify the role of KIAA1522 in lung adenocarcinomas, and the molecular events that underlie KIAA1522-mediated chemoresistance to the platinum.

**Methods:**

Immunohistochemistry were used to detect KIAA1522 expression in clinical NSCLC samples. Then, the survival analyses were performed to assess the link between KIAA1522 expression and overall survival or therapeutic outcome. In vivo depletion of KIAA1522 in adenocarcinoma cells were achieved by adeno-associated virus-mediated sgRNA/Cre delivery into the conditional Kras^G12D^/Cas9 expressed mice, which were designated to identify the roles of KIAA1522 in tumorigenesis and/or chemotherapy responses. The effects of KIAA1522 and downstream molecular events were studied by pharmacology in mice model and assays using in vitro cultured cells. The clinical relevance of our findings was examined by data-mining of online datasets from multiple cohorts.

**Results:**

The clinical evidences reveal that KIAA1522 independently predicts both the overall survival and the outcome of platinum-based chemotherapy in lung adenocarcinomas. By using a *Kras*^*G12D*^-driven murine lung adenocarcinoma model and performing in vitro assays, we demonstrated that KIAA1522 is a critical positive regulator of lung adenocarcinoma and a modulator of cisplatin response. KIAA1522 potentiates the TNFα-TNFR2-NFκB signaling which in turn intensifies recalcitrance to cisplatin treatment. These results were further manifested by integrative bioinformatic analyses of independent datasets, in which KIAA1522 is tightly associated with the activity of TNFα-NFκB pathway and the cisplatin-resistant gene signatures. More strikingly, overexpression of KIAA1522 counteracts the cisplatin-induced tumor growth arrest in vivo, and this effect can be remarkably diminished by the disruption of NFκB activity.

**Conclusion:**

High expression of KIAA1522 is turned out to be an indicator of dismal effectiveness of platinum-based therapy in lung adenocarcinomas. KIAA1522 hyperactivates TNFα-NFκB signaling to facilitate resistance to platinum reagents. Targeting NFκB signaling through small molecule inhibitors may be a rational strategy to conquer chemoresistance and synergize platinum-based chemotherapy in KIAA1522 overexpressed lung adenocarcinomas.

## Background

Lung cancer is the most common type of human malignancy and causes more cancer-related mortality than other diseases worldwide [1]. Non-small cell lung cancer (NSCLC) is the major histological subtypes of lung cancer, which is comprised by lung adenocarcinoma (ADC) and lung squamous cell carcinoma (SCC) [2]. Several therapeutic advances have been achieved in recent years, especially the progress in target therapy and the emergence of immunotherapy [3, 4]. However, a limited number of subtypes are benefited from these two methods and the overall survival rates of NSCLC remain low. Curative surgery is the preferred choice for the majority NSCLC patients in early and late stage. Unfortunately, even the NSCLC patients in the advanced stage underwent complete resection, about 70% patients have dismal prognosis due to tenacious drug resistance [2, 5]. Nowadays, platinum-based chemotherapy remains the cornerstone of routine adjuvant chemotherapy. This approach moderately improves 5-year survival rate, whereas its long-term clinical effectiveness severely impeded by the inherited or acquired resistance to the platinum-based reagents [6–8].

Cisplatin is one of the most widely used and studied platinum-based cytotoxic drugs. But none of specific biomarker that guide cisplatin usage in the clinical practice. Also, there is still lack of effective reagents overcoming cisplatin-resistance. It has been known that some genetic variations and alterations of key signaling pathways can enhance cisplatin insensitivity [9]. NFκB signaling is one of the crucial signaling pathways associated with chemoresistance [10–12]. Hyperactivation of NFκB signaling is implicated in multiple types of cancers, contributing to tumor initiation, development, progression and responses to extracellular stimulations [13]. The transcription factor NFκB is a pleiotropic hetero- or homo-dimer, complexed by RelA/p65, RelB, p52, p50 or c-Rel. The pro-inflammatory cytokine TNFα is a robust upstream regulator of NFκB. Once stimulated by TNFα, the TNF receptors recruit certain cytoplasmic proteins to form an IKK regulating complex to active IKKα/β, then IKKα/β phosphorylates IκB and unleashes p50: p65 complex into nucleus. The activation of NFκB transcriptional factor regulates the expression of certain anti-apoptotic genes, such as XIAP, BCL-2 and BIRC5/survivin, which counteract the cytotoxic effects of cisplatin [14–16]. Notably, there is ample evidence supporting that NFκB is essential for the development and chemoresistance of lung adenocarcinoma [17–20].

The functions of KIAA1522 was not investigated until the recent years. It was reported as an early-diagnostic biomarker in one of our previous work [21]. Furthermore, we found that KIAA1522 promotes NSCLC development via the RAS-MEK-ERK pathway [22]. Otherwise, KIAA1522 accelerates the metastatic ability of esophageal carcinoma cells and breast cancer cells [23, 24]. Here, we describe an unappreciated role of KIAA1522 in potentiating TNFα-NFκB signaling, and thereby give rise to cisplatin resistance. Also, we will explore several research questions: whether KIAA1522 contributes to chemoresistance in experimental lung cancer models. What are the potential mechanisms responsible for KIAA1522-induced chemoresistance? And how can we restore sensitivity to platinum-based therapy in KIAA1522 high expressed ADC samples. These efforts may suggest a future avenue for patients’ treatment.

## Methods

### Patients and samples

The NSCLC samples were procured in the Cancer Hospital, Chinese Academy of Medical Sciences and Peking Union Medical College (CAMS & PUMC). Primary tumor tissues and adjacent non-tumoral lung tissues were excised and pathological diagnoses by experienced pathologists. The tissue samples were fixed with neutral buffered formalin (pH 7.4) and paraffin-embedded for the construction of tissue microarrays. All of the tissues were residual specimens after diagnostic sampling. None of the patients received pre-surgical treatment. The basic clinicopathologic data were listed in **Table S1**. This study was approved by the Ethics Committee/ Institutional Review Board of the Cancer Institute (Hospital), PUMC/CAMS (No. 12–098/632). Written informed consent forms were obtained from patients for sampling and research. And all the methods in our study were carried out in accordance with the approved guidelines.

### Cell culture

The human lung adenocarcinoma cell lines A549 and NCI-H1299 were acquired from the American Type Culture Collection (ATCC, Manassas VA, USA). The murine lung cancer cell line 889-DTC (889) was a kindly provided by professor Winslow [25, 26]. HEK293T cells were acquired from the American Type Culture Collection (ATCC, Manassas, VA, USA). Cell lines were maintained at 37 °C in 5% CO2 in Dulbecco’s modified Eagle medium supplemented with 10% fetal bovine serum.

### Antibodies and reagents

Recombinant human TNFα (AF-300-01A) was purchased from PeproTech. Cisplatin (HY-17394) was from MedChemExpress, QNZ (S4902) and Cycloheximide (CHX) was from Selleck. The primary antibodies used in this work are as follow: KIAA1522 (WB, 1:1000; IHC, 1:200; HPA032050, Sigma ImmunoChemicals, St Louis, MO, USA), Phospho-NF-kB p65 (Ser536) (WB, 1:1000; CST, 3033); NF-κB p65 (WB, 1:1000; CST, 8242), Phospho-IkBα (Ser32) (WB, 1:1000; CST, 2859), IkBα (WB, 1:1000; CST, 4814), Phospho-IKKα/β (Ser176/180) (WB, 1:1000; IHC, 1:150; CST, 2697), IKKα (WB, 1:1000, CST, 11930), TNFR1 (WB, 1:1000, Proteintech, 21,574–1-AP), TNFR2 (WB, 1:1000; IHC, 1:50, Proteintech, 19,272–1-AP), GAPDH (WB, 1:1000, Santa Cruz, sc-25,778), β-actin (WB, 1:2000, Santa Cruz, sc-47,778), Ki67 (IHC, 1:200, GeneTex, GTX16667), cleaved caspase-3 (IHC, 1: 500, CST, 9664).

### Plasmid and lentivirus package

C-terminal Flag-tagged KIAA1522 were cloned into pLVX-IRES-ZsGreen plasmid. The forward shRNA sequences for targeting human KIAA1522 are as follow: sh-1: CCGGCCACGGCCTTCGATACTTATGCTCGAGCATAAGTATCGAAGGCCGTGGTTTTTG; sh-2: CCGGCCTACCTGTCGAAGTTGATTCCTCGAGGAATCAACTTCGACAGGTAGGTTTTTG. shRNA sequences were cloned into pLKO.1 plasmid (Sigma-Aldrich, Missouri, USA). The sgRNAs for targeting mouse kiaa1522 gene are as follow: sg-1: GCCGAGAGTGACAACCGTCA; sg-2: AGTGGGAGACCTCCTCATCT. The sgRNA sequences were cloned into lentiCRISPRv2 plasmid which was a gift from Feng Zhang (Addgene plasmid # 52961) [27] The lentiCRISPR-EGFP sgRNA1 plasmid (Addgene plasmid #51760) from Feng Zhang was used as sgRNA control [28]. HEK293t cells were used for lentivirus packaging. Lentiviral vectors expressing target genes were co-transfected with lentiviral packaging plasmids psPAX2 (Addgene plasmid, 12,260) and pMD2.G (Addgene plasmid, 12,259) with Lipofectamine® 3000 (Invitrogen). The medium containing lentivirus was harvested at 48 h and 72 h after transfection and used to infect cultured cell lines. Transduced cells were isolated by puromycin selection or FASC sorting.

### Adeno-associated virus (AAV) production and mice management

The sgRNA sequences for AAV-mediated in vivo kiaa1522 editing are GGAAGTCAGGAAGGCGACGG and GCCGAGAGTGACAACCGTCA. Each sgRNA was connected next to a U6 promoter and serially cloned into the pAAV-U6-gRNA v2.0-CMV-NLS-Cre-3FLAG-P2A-EGFP-WPRE vector (OBiO Technology (Shanghai) Corp., Ltd), which express a nuclear localization signal fused Cre protein. The sgkiaa1522-expressing AAV was generated by co-transfection of HEK293T cells with AAV expression vector, AAV helper plasmid and AAV Rep/Cap plasmid. The cells were lysed by a freeze-thaw procedure at 72 h after transfection. The viral particles were purified by iodixanol step-gradient ultracentrifugation and concentrated by a molecular-mass-cutoff ultrafiltration device. a non-sense sequence GCACTACCAGAGCTAACTCA was also cloned into the pAAV vector to generated the sg-control virus. The B6-Gt (ROSA)26Sor^tm1(CAG-LSL-cas9,-tdTomato)/^Nju (LSL-Cas9) mice (from Nanjing University Model Animal Resource Platform) were crossed by *Kras*^*(LSL-G12D)*^ mice (from Shanghai Model Organisms Center, Inc.) to generate the conditionally Cas9/ Kras^G12D^ expressing mice. 8–12-week-old mice were anesthetized by intraperitoneally injection of pentobarbital sodium, and then delivered intratracheally by the recombinant AAV particles (2 × 10^11^/mouse). For in vivo cisplatin treatment, the mice were treated intraperitoneally by cisplatin (3.5 mg/kg B.W.) once a week for one month.

### Allograft/xenograft tumor model and in vivo pharmacology

For in vivo tumorigenesis assays, 5 × 10^5^ 889 cells were subcutaneously injected into C57BL/6 mice (5–6 weeks age) for 1 month. 5 × 10^6^ A549 cells were subcutaneously injected into BALB/c nude mice (5–6 weeks age) for 2 months. The control and KIAA1522-overexpressed A549 cells were subcutaneously injected into C57BL/6 mice. Two weeks later, the mice were subjected to drug treatment: cisplatin (7 mg/kg B.W.) once a week and QNZ (1 mg/kg B.W.) twice a week via intraperitoneal injection. The tumor volumes were measured by the formula: (length×width^2^)/2.

### Colony formation assays

To assess the inhibitory effects of small-molecular reagents in vitro, 1 × 10^4^ cells were seeded in 12-well plates. 72 h later the cells were treated with vehicle, cisplatin (10–20 μM), QNZ (500 nM), or combination for 48 h. Then the cells were fixed by methanol and stained with crystal violet (Beyotime, C0121).

### Cell viability analysis

Cells were seeded at 2000 cells in 200 μL DMEM per well in 96-well culture plates. At the indicated time points, 20 μl Cell Counting Kit-8 reagent (Beyotime, C0039) was added to each well and incubated at 37 °C for 2–4 h. The absorbance values (OD 450 nm) were measured using a spectrophotometer. The absorbance values at 600 nm were used as references.

### Luciferase reporter assay

889 cells with or without TNFα (10 ng/ml) treatment were transiently transfected with NFκB luciferase reporter plasmid (1 μg), and 20 ng of the Renilla luciferase plasmids. 48 h post-transfection, the firefly and Renilla luciferase activities were monitored using the Dual-Luciferase Reporter Assay System (Promega). NFκB signaling activity is determined by the ratio of firefly to Renilla luciferase activity.

### Western blot and co-immunoprecipitation

Cells were lysed by RIPA buffer (Thermo Fisher Scientific, 89,901) with protease inhibitors cocktail (Roche Diagnostics, 05892970001) and phosphatase inhibitor cocktail (Roche Diagnostics, 04906845001). The lysates were clarified by centrifugation at 13000 g for 30 min at 4 °C. Protein concentrations were determined by BCA protein assay kit (Thermo Fisher Scientific, 23,225) followed by boiled with loading buffer. Protein samples (50–150 μg) were separated through SDS-PAGE, then transferred to nitrocellulose filter membrane (Pall Corporation) blocked and incubated with the primary antibodies. After washing with TBST, the blots were incubated with Anti-rabbit IgG HRP-linked antibody (CST, 7074) and Anti-mouse IgG HRP-linked antibody (CST, 7076), then visualized by the SuperSignal West Dura Extended Duration Substrate (Thermo Fisher Scientific, 34,076).

Co-immunoprecipitation (Co-IP) was performed using Protein G–agarose suspension (Millipore, 16–266). The cells were lysed by IP lysis buffer (Beyotime Institute of Biotechnology, P0013), and then incubated by 50 μl of Protein G–agarose suspension for 3 h at 4 °C on a rocking platform to reduce non-specific binding. After removing the beads, the supernatant was supplemented with the primary antibodies followed by incubation for another 3 h at 4 °C. A total of 100 μl of Protein G–agarose was then mixed to each sample, and the incubation was continued overnight on a rocking platform. The immunoprecipitates were collected by centrifugation and washed three times with the TBS. The agarose was boiled with loading buffer and subjected to western blot analysis.

### Immunohistochemistry

The tissue microarrays and slides were deparaffinized, rehydrated, immersed in 3% hydrogen peroxide solution for 15 min, heated in citrate buffer (pH 6.0) for 25 min at 95 °C, and cooled for at least 60 min at room temperature. Between each incubation step, the slides were washed three times with PBS (pH 7.4). After blocked with 10% normal goat serum for 30 min at 37 °C and washed, the slides were incubated overnight at 4 °C with primary antibodies against target proteins and visualized using the PV-9000 Polymer Detection System (GBI, USA) following the manufacturer’s instructions or GTVisionTMIII Detection System/Mo&Rb (GeneTech, GK500710). After washing with PBS, the slides were counterstained with hematoxylin.

### Immunohistochemical assessment and analysis

Protein expression levels were determined on the basis of staining intensity and the percentage of immunoreactive cells. Staining intensity was rated as 0 (negative), 1 (weakly positive), 2 (moderately positive), and 3 (strongly positive). The percentage of immunoreactive cells was graded as 0 (0%), 0.5 (1–10%), 1 (11–20%), 2 (21–50%), 3 (51–80%), or 4 (81–100%). The average of tumor cell staining intensity score multiplied by the percentage of positive cells score represented the final score of one sample. The prognostic value of certain protein was evaluated by univariate or multivariate cox regression analyses in different subtypes of patients using R packages survival and survminer. The Nomogram survival predictive model was constructed using RMS package. The optimal cutoff value of IHC staining scores were estimated by R package maxstat.

### RNA sequencing

Total RNA was extracted by TRIZOL Reagent (Life technologies, CA, USA) according to the manufacturer’s instructions, then checked for RIN numbers to inspect RNA integrity by Agilent Bioanalyzer 2100 (Agilent technologies, CA, USA). Qualified total RNA was further purified using RNAClean XP Kit (Beckman Coulter, Inc. CA, USA) and RNase-Free DNase Set (QIAGEN, GmBH, Germany).

Library construction and sequencing were performed at the Shanghai Biotechnology Corp. RNA libraries were prepared for sequencing using VAHTS Universal V6 RNA-seq Library Prep Kit for Illumina (Vazyme, Nanjing, China) before submitted to Illumina Hiseq 2500 system. Clean data was generated by trimming the adaptor and filtering rRNAs using Seqtk (https://github.com/lh3/seqtk). Then the clean reads were mapped to mouse reference genome (GRCm38) with Hisat2 (version: 2.0.4) to generate the BAM files for each sample. The uniquely mapped fragments of genes were counted by Stringtie (version:1.3.0). Gene expression was evaluated by normalized Fragments Per Kilobase of exon model per Million mapped reads (FPKM) using TMM (trimmed mean of M values) methods. The raw data of RNA sequencing were deposited in Gene Expression Omnibus (GEO accession number: GSE146072).

### Bioinformatic analyses of RNA sequencing results

The bioinformatic analyses were completed through R programming language (version 3.6.2) in RStudio software. In the processed RNA-seq data, the genes with more than 500 total counts were subjected to the edgeR analysis to estimate the fold change of each gene between groups. Under the criterion that FDR q-value≤0.05, and log2FC ≥ 1 or ≤ − 1. The pathway enrichment analysis and the gene-sets enrichment analysis (GSEA) were performed using ClusterProfiler package [29]. The gene expression matrix was transformed to an enrichment score matrix by GSVA package [30] for the comparison of different genesets between groups. The genes significantly down-regulated by KIAA1522-depletion in 889 cells were defined as the KIAA1522 positive regulated genes which were constructed to be a geneset by GSEABase package and subjected to the following data mining analyses.

### Integrative transcriptome analyses of multi-central datasets

We downloaded FPKM-normalized RNA-seq data of TCGA-LUAD and TCGA-LUSC datasets together with the associated sample information using TCGAbiolinks package [31]. The FPKM values were transformed to TPM values for the following calculation. The microarray-derived transcriptome datasets from GEO database were downloaded by GEOmirror package and the sample information were acquired by GEOquery package. The lung datasets include GSE3141, GSE8894, GSE13213, GSE11969, GSE37745, GSE31210, GSE30219, GSE50081, GSE43580 and GSE14814. For the GEO datasets including more than one histological type, the lung adenocarcinoma samples were selected for further usage. The single cell sequencing dataset E-MTAB-6149 [32] was downloaded from the ArrayExpress website. The data was processed by Seurat package [33] for tSNE dimension reduction and clustering. GSVA program was used to determine KIAA1522 signature score and the scores of genesets associated with TNFα-NFκB signaling and cisplatin resistance. Correlation analyses were performed by cor.test function, and visualized by ggpubr package. Datasets containing survival information were used to perform survival analyses and cox regression analyses by survival and survminer package. Cutoff values for the tested factors were estimated by maxstat package. Meta-analyses were performed using metafor package in the fixed-effects model and the visualization was achieved by either forestplot or ggplot2 package. Alluvial diagram was drawn by alluvial package.

### Statistical analysis

Statistical analyses were conducted by GraphPad Prism 8.0 software and R statistical packages version 3.6.2. Significant differences between groups were examined through student’s t-test. The Kaplan–Meier curves were tested by Log-rank test. All *P* values < 0.05 were considered significant.

## Results

### High expression of KIAA1522 indicates resistance to platinum-based chemotherapy in lung adenocarcinoma

**Fig. 1:**
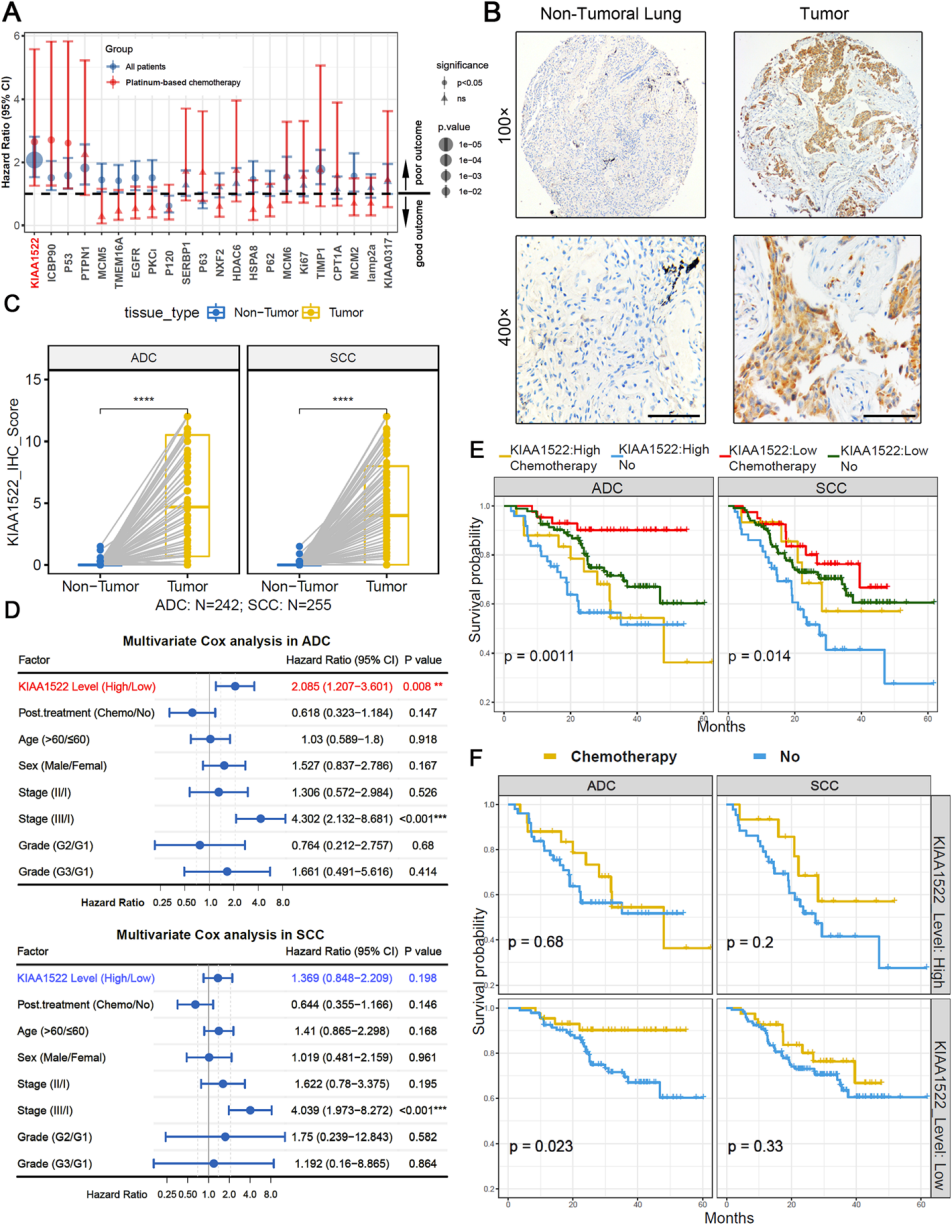
KIAA1522 expression predicts platinum-based therapeutic effectiveness in lung adenocarcinomas. **a** Forest plot shows the hazard ratio with 95% confidence interval of each tested protein. The hazard ratios are estimated by univariate cox analyses in all NSCLC patients (blue) or the patients receiving post-surgery platinum-based chemotherapy (red). The proteins were ranked by the *p*-values in the chemotherapy group. **b-f** Immunohistochemical analyses of KIAA1522 expression in tissue samples from NSCLC patients. **b** Representative images of KIAA1522 immunohistochemical staining in NSCLC specimens and non-tumoral normal lung tissues. Bar = 100 μm. **c** Paired comparisons of KIAA1522 expression in non-tumoral and tumor samples in either lung adenocarcinomas (ADC) or squamous cell carcinomas (SCC), paired t-test, ****P < 0.0001. **d** In ADC and SCC patients, multivariate cox analyses of prognostic values of KIAA1522 levels excluding age, sex, stage, grade and post-surgical chemotherapy. **e** Kaplan–Meier curves show the overall survival of NSCLC patients expressing high/low levels of KIAA1522 protein with or without platinum-based post-surgical adjuvant chemotherapy. **f** Comparisons of prognostic effects of platinum-based chemotherapy in KIAA1522 high expression or low expression groups respectively

Platinum-based adjuvant chemotherapy is the mainstay of post-surgery management for non-small cell lung carcinoma (NSCLC). However, a group of patients experienced drug insensitivity. To identify key genes driving and predicting chemoresistance. We initially screened our previously identified candidate NSCLC biomarkers [21, 34] for their prognostic value in the patients receiving platinum-based chemotherapy. After preliminary immunochemical assays in a few paired NSCLC and non-tumoral tissues, there were twenty-two candidate expression-altered proteins that were tested in a total of 598 primary NSCLC samples plus 500 adjacent non-tumoral lung tissues dissected from 598 patients, showing extremely high expression of these proteins in tumoral specimens (Supplementary Fig. 1A). Univariate cox analyses were used to examine their association with chemotherapeutic outcome, illustrating that the predictive effectiveness of KIAA1522 were more predominant than other proteins (Fig. 1a). Multivariate cox analysis demonstrated that KIAA1522 was an independent prognostic factor in NSCLC, in either patients receiving chemotherapy or all NSCLC patients (Supplementary Fig. 1B). Similarly, in a nomogram predictive model of NSCLC patients, expression levels of KIAA1522 contributed to lower survival rates (Supplementary Fig. 1C-D).

Immunohistochemistry assays in paired tumoral and non-tumoral tissues showed that KIAA1522 was significantly elevated in both adenocarcinoma and squamous cell carcinoma (Fig. 1b-c). To distinguish the chemo-sensitive predictive roles of KIAA1522 in different histological subtypes. We performed multivariate cox analyses in adenocarcinoma and squamous cell carcinoma separately. The results revealed that KIAA1522 displayed significant effect in adenocarcinoma patients but not in squamous cell carcinoma (Fig. 1d). In our cohort of NSCLC patients, there is no difference in survival rate between histological types in both chemo-naive and chemotherapy-experienced patients (Supplementary Fig. 1E). The platinum-based chemotherapy seems to improve the survival probability, but hardly separate the Kaplan–Meier curves significantly (Supplementary Fig. 1F). Remarkably, in both ADC and SCC, incorporating the expression levels of KIAA1522 successfully stratified one group of lung adenocarcinoma patients with the most favorable outcomes, who express lower level of KIAA1522 and underwent platinum-based chemotherapy (Fig. 1e). When the NSCLC patients were grouped by histological types and KIAA1522 levels, chemotherapy in KIAA1522-low expression group of adenocarcinomas was more efficient than other groups (Fig. 1f). In TCGA lung cancer datasets, we found that the expression of KIAA1522 elevated in both adenocarcinomas and squamous cell carcinomas (Supplementary Fig. 2A), but only in the adenocarcinoma datasets, KIAA1522 expression connected with poor prognosis (Supplementary Fig. 2B). Above all, the clinical analyses highlight the predictive value of KIAA1522 in both overall survival and survival after platinum-based chemotherapy. Meanwhile, the results suggest the tumor-promoting and chemo-resisting roles of KIAA1522 in lung adenocarcinomas.

### Depletion of KIAA1522 impairs tumorigenesis and potentiates chemosensitivity in *Kras*^*G12D*^-induced murine lung adenocarcinomas

**Fig. 2:**
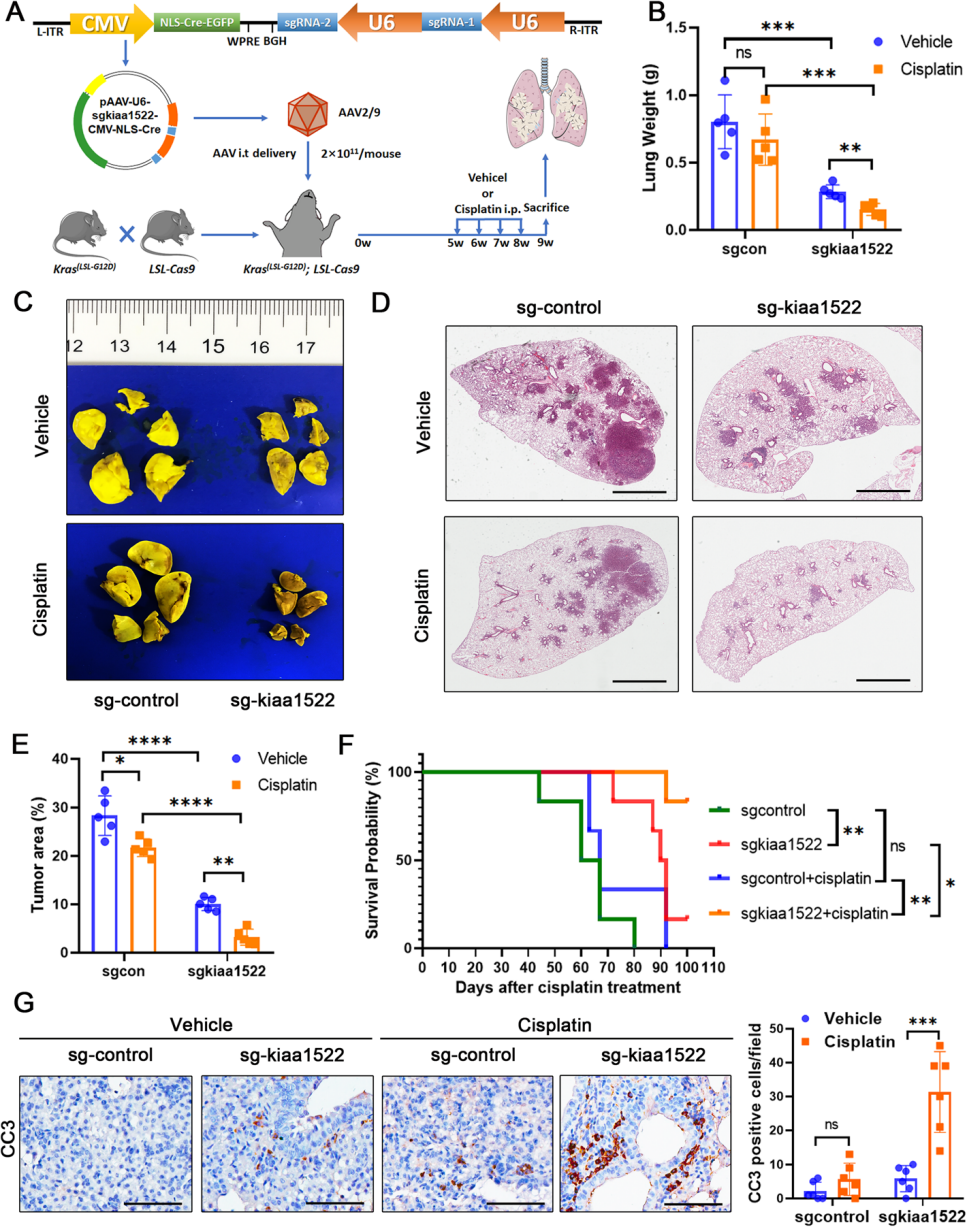
Genetically depletion of kiaa1522 gene impairs *Kras*^*G12D*^-induced lung adenocarcinoma and sensitizes tumors to cisplatin. **a** Schematic showing of adenovirus associated virus (AAV)-mediated kiaa1522 depletion in *Kras*^*G12D*^-induced lung. The *Kras*^*(LSL-G12D)*^, *LSL-Cas9* mice were intratracheal administrated by Cre- and control sgRNA or kiaa1522 sgRNAs-expressing-AAVs. Then, the mice were subjected to i.p. injections of vehicle/cisplatin. **b** The comparison of lung weights in different groups. t-test, **P < 0.01, ***P < 0.001. **c**-**d** Representative image of the lung after Bouin′s staining (**c**) and HE staining (**d**) in each group. **e** The comparison of tumor area in different groups. t-test, *P < 0.05, ****P < 0.0001. Bar = 2 mm. **f** Kaplan-Meier curves show the role of KIAA1522-depletion and/or effect of cisplatin on the outcome of lung cancer in mice. The survival rates were compared between each group. Log-rank test, *P < 0.05, **P < 0.01. **g** Immunohistochemical staining of cleaved-caspase 3 in the lung tumor samples from the indicated mice. t-test, ***P < 0.001. Bar = 100 μm

We employed an oncogenic Kras-induced lung adenocarcinoma model to study whether and to what extend KIAA1522 influence tumorigenesis and chemoresistance. The kiaa1522 specific sgRNAs were integrated together with a Cre expressing cascade into recombinant adeno-associated virus (AAV). Then, the rAAVs were delivered intratracheally to the *LSL-Cas9/LSL-Kras*^*G12D*^ mice (Fig. 2a). The mice delivered by control sgRNA and kiaa1522 sgRNA were divided into two groups treated by vehicle and cisplatin respectively (Fig. 2a). After the mice were sacrificed, the lungs were weighted (Fig. 2b), then subjected to Bouin′s staining or HE staining (Fig. 2c). The tumoral lungs in the kiaa1522 sgRNA-introduced mice had reduced lung weights compared to control mice. Upon cisplatin management, the lung weights were further lost in the kiaa1522-edited mice (Fig. 2b). When comparing the tumor burden between groups, we found that down-regulation of KIAA1522 dramatically attenuated *Kras*^*G12D*^-induced lung adenocarcinoma in situ. More importantly, the depleting of KIAA1522 synergistically improved the efficiency of cisplatin in shrinking lung tumors (Fig. 2c-e, Supplementary Fig. 2C). The following survival analysis also substantiated the benefits of KIAA1522 down-regulation which not only elevated the survival rate of tumorigenic mice but also made them more sensitive to cisplatin therapy (Fig. 2f). Coincidently, the combination of KIAA1522 depletion and cisplatin treatment considerably induced apoptosis in vivo, monitored by immunohistochemical staining of cleaved-caspase 3 (Fig. 2g).

**Fig. 3:**
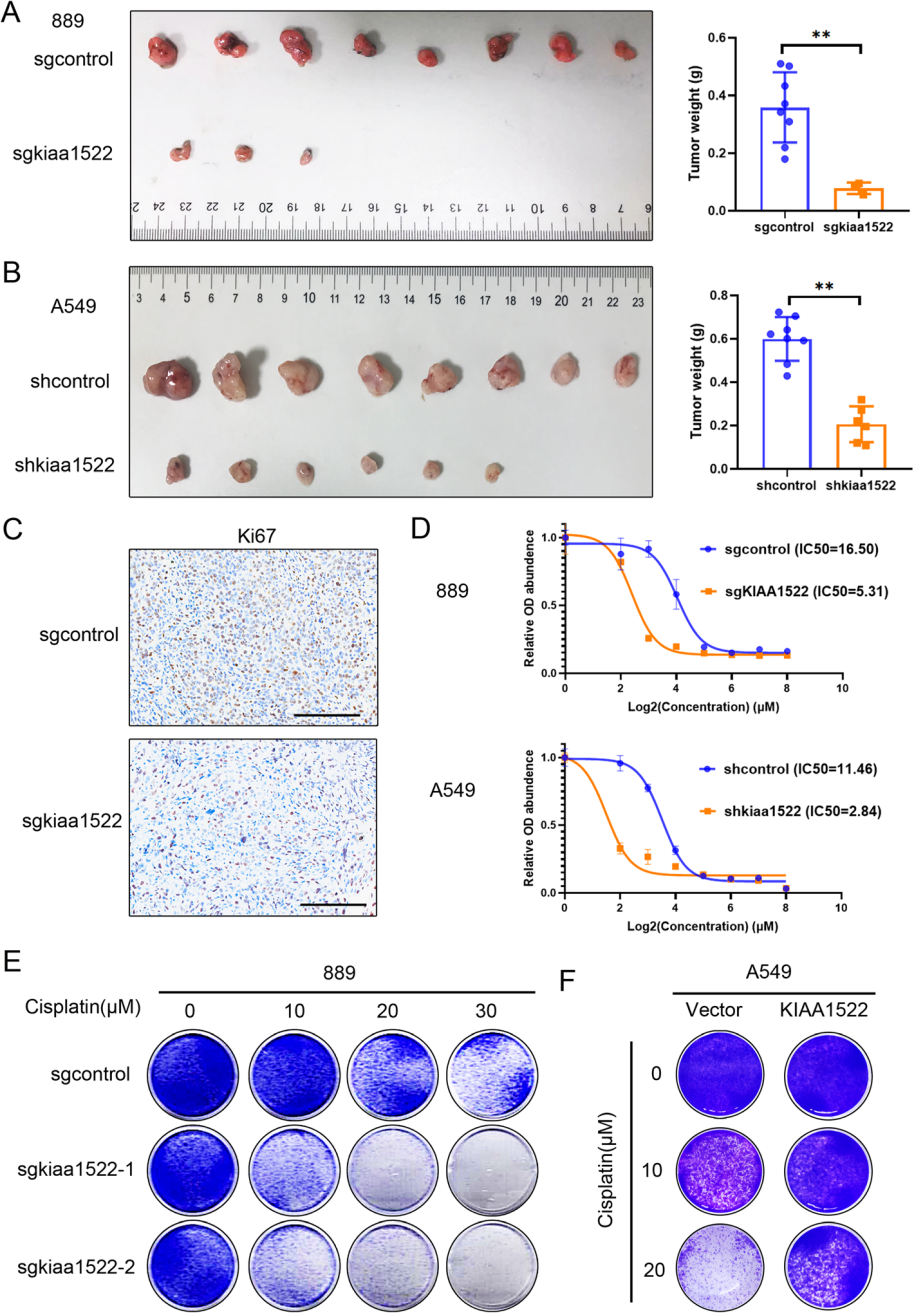
Depletion of KIAA1522 in lung adenocarcinoma cells reduces tumor growth and resistance to cisplatin. **a** Murine cell line 889 with the sgRNA-mediated depletion of KIAA1522 were injected into C57BL/6 J mice subcutaneously. One month later, the mice were sacrificed and the tumor weights were quantified. t-test, **P < 0.01. **b** Human adenocarcinoma cell A549 expressing KIAA1522-shRNA were injected into nude mice subcutaneously. After two months, the mice were subjected to tumor weights quantification. t-test, **P < 0.01. **c** Immunohistochemical staining of Ki67 in control sgRNA/Cas9 or kiaa1522-sgRNA/Cas9 expressing 889 cells. Bar = 100 μm. **d** IC50 of cisplatin in both 889 cells and A549 cells with down-regulated KIAA1522 were analyzed by CCK8 assays. **e**-**f** The inhibitory effects of cisplatin on 889 cells expressing kiaa1522-sgRNA/Cas9 (**e**) or A549 cells overexpressing KIAA1522 (**f**) by colony formation assays

We also down-regulated KIAA1522 expression in the established lung adenocarcinoma cell lines and analyzed by their tumorigenic capability and cisplatin response. The results showed that loss of KIAA1522 severely impaired subcutaneous tumors (Fig. 3a-b), and reduced the amount of Ki67 positive cells in vivo (Fig. 3c). Moreover, down-regulation of KIAA1522 reduced the IC50 value in response to cisplatin (Fig. 3d). The KIAA1522 depleted cells were more sensitive to cisplatin treatment in vitro (Fig. 3e). Oppositely, overexpression of KIAA1522 rendered the cells chemoresistance to cisplatin (Fig. 3f).

The above results strongly indicate that KIAA1522 is required for both the tumorigenesis and resistance to cisplatin in lung adenocarcinoma, these facts coincide with the clinical relevance of KIAA1522 in lung adenocarcinoma patients.

### KIAA1522 promotes the TNFα-NFκB signaling pathway

**Fig. 4:**
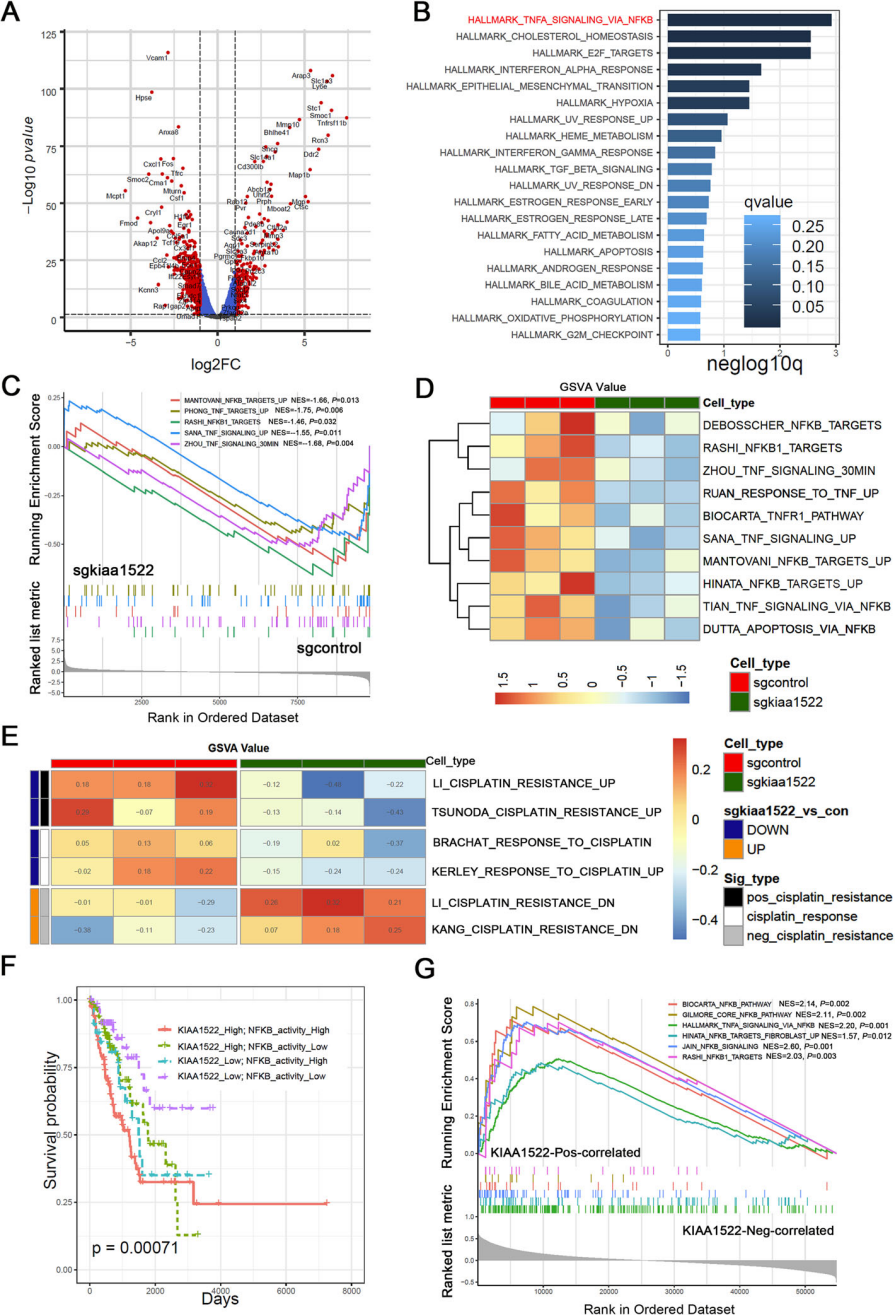
KIAA1522 regulates gene expression signatures associated with TNF-NFκB pathway and cisplatin resistance. **a**-**e** RNA-sequencing experiments were performed to detect the expression profiling of control and 889 cells depleting of KIAA1522. Each group includes three replicates. **a** volcano plot shows the different expressed genes of KIAA1522 depleted 889 cells compared to the control cells. The genes with both |log_2_FC| > 2 and p-value< 0.05 were considered as significantly differentiated genes. **b** Enrichment analysis of KIAA1522 positively regulated genes in HALLMARK gene signatures, the significantly enriched signatures were ranked by q-values, the bar plot shows the top 20 signatures. **c** Gene sets enrichment analysis (GSEA) of genes by ranked by log_2_FC (sgkiaa1522/ sgcontrol) values using a set of TNF-NFκB pathway related signatures. **d**-**e** The GSVA score of the genesets related to the TNF-NFκB pathway (**d**) and experiments derived cisplatin-resistant gene signatures (**e**) were determined by GSVA algorithm. The heatmaps show the distribution of GSVA values between the indicated group of cells. **f** The patients in TCGA-LUAD cohort were classified by both KIAA1522 expression and NFκB activity into four groups and subjected to Kaplan–Meier overall survival analysis. The activity of NFκB signaling was determined by the GSVA score of the geneset JAIN_NFKB_SIGNALING. **g** The genes were ranked by the Pearson correlation coefficient with KIAA1522 expression in TCGA-LUAD datasets. Then, the GSEA assays were performed using a set of NFκB activity positively associated signatures to evaluate the correlation of KIAA1522 expression level with NFκB signaling activity

To understand the molecular basis of KIAA1522-mediated acquirement of chemoresistance to cisplatin, we performed transcriptional profiling assays to detect dysregulated genes upon KIAA1522 depletion in 889 lung adenocarcinoma cells (Fig. 4a). Enrichment analysis of KIAA1522 positive regulated genes using HALLMARK signatures showed that the geneset HALLMARK_ TNFA_ SIGNALING_ VIA_ NFKB pathway was on the top of enriched pathways (Fig. 4b), suggesting that KIAA1522 may enhance TNFα-NFκB signaling pathway. This conclusion was further strengthened by gene set enrichment analysis (GSEA) and gene set variation analysis (GSVA) analysis. GSEA and GSVA results both showed that TNFα-NFκB signaling associated genesets were down-regulated in the KIAA1522 depletion cells (Fig. 4c-d). Similar to TNFα-NFκB signatures, The GSVA scores of cisplatin resistance signatures and cisplatin-responsive signatures were also down-regulated in the 889 cells expressing sg-kiaa1522. While the gene signatures negatively related to cisplatin resistance were up-regulated in KIAA1522 down-regulated cells (Fig. 4e).

In the TCGA-LUAD dataset, when considering KIAA1522 expression (KIAA1522 mRNA) and TNFα/NFκB status together, the patients with low KIAA1522 and TNFα signal levels or low KIAA1522 and NFκB activity had the best outcomes (Supplementary Fig. 3A; Fig. 4f). In addition, we also observed the correlation of KIAA1522 expression with both TNF signaling (Supplementary Fig. 3B), and NFκB activity (Fig. 4g) in the GSEA assays. TNFα-NFκB signaling has been reported to cause cisplatin insensitivity in several types of malignancies [13]. We found that inhibition of KIAA1522 reduced a set of genes down-stream of NFκB signaling, including anti-apoptotic genes confering cisplatin resistance, such as Xiap, Bcl2 and Birc5/survivin [15] (Supplementary Fig. 3C). So, the hyperactivation of TNFα-NFκB signaling may be a key molecular event responsible for KIAA1522-induced counteractivity to cisplatin.

**Fig. 5:**
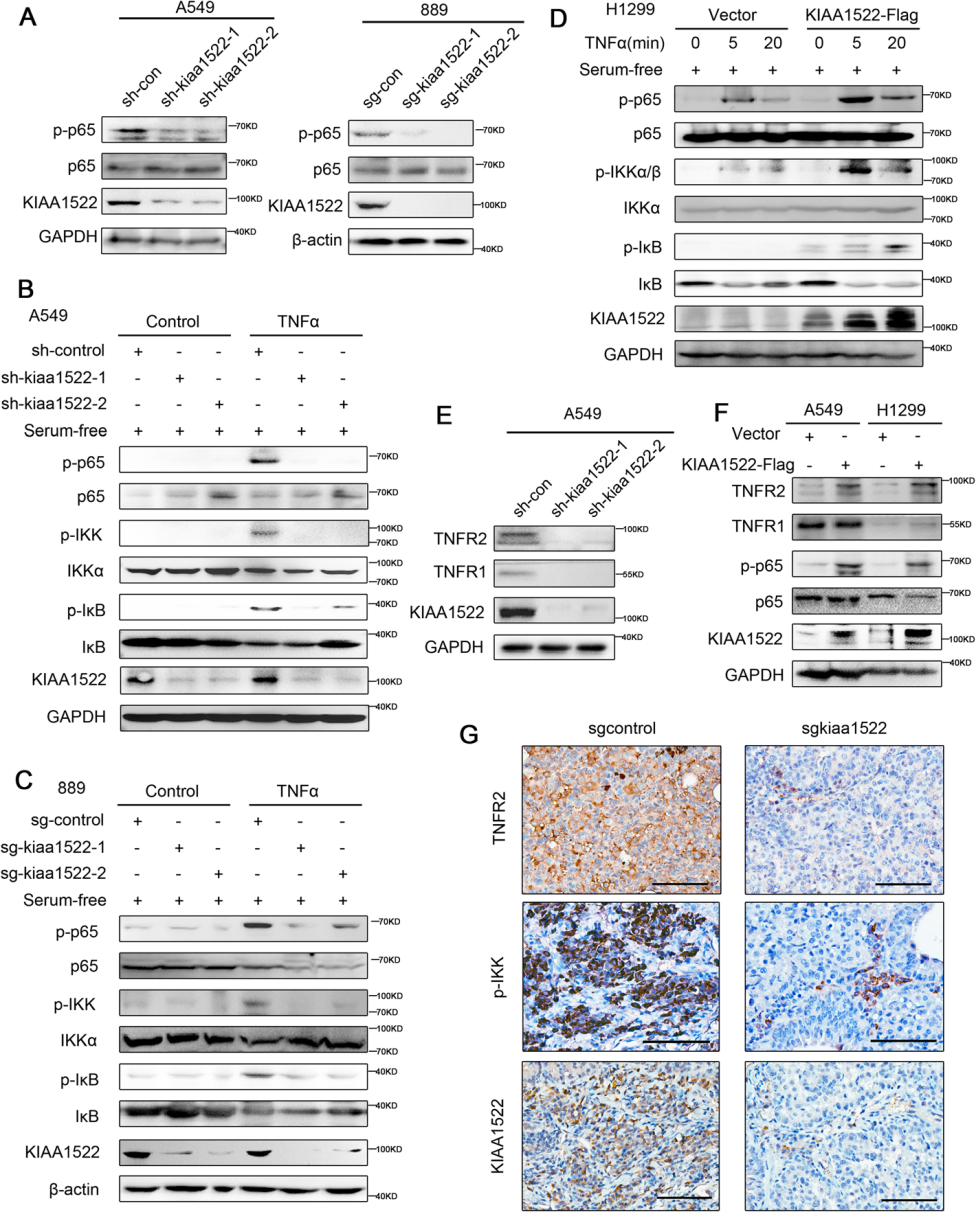
KIAA1522 positively regulates TNFα-NFκB signaling in lung adenocarcinoma cells. **a** Immunoblotting assays of Phospho-NFκB p65 (Ser536) in the control and KIAA1522 down-regulated cells. **b**-**c** The indicated group of A549 cells (**b**) or 889 cells (**c**) were deprived of serum for 20 h, then treated with TNFα for 5 min. Then the cells were tested by Western blotting assay to detect the phosphorylation of p65, IKK and IκB proteins. **d** H1299 cells overexpressing Flag tagged KIAA1522 protein were cultured in serum-free condition for 20 h, then treated by TNFα and harvest at the indicated time point. The harvested cells were subjected to Western blotting assay. **e** Western blotting assays show the expression of TNFR2 and TNFR1 in KIAA1522 down-regulated A549 cells. **f** Western blotting assays show the expression of TNFR2 and TNFR1 in KIAA1522-overexpressed cells. **g** Immunohistochemical staining of TNFR2, phosphorylated IKKα/β and KIAA1522 in *Kras*-induced murine lung tumor samples with or without KIAA1522 depletion. Bar = 100 μm

We next explore whether KIAA1522 promotes TNFα-NFκB signaling transduction in the cultured cells. We found that down-regulation of KIAA1522 by shRNA or Cas9/sgRNA substantially inhibited the basal levels of phosphorylated NF-κB p65/RelA (Fig. 5a, Supplementary Fig. 4A). In the presence of exogenous TNFα to stimulate NFκB signaling in the serum-deprived cells, loss of KIAA1522 remarkably alleviated NFκB signaling activity monitored by luciferase reporter (Supplementary Fig. 4B). Western blotting assays showed that TNFα was less effective to up-regulated the phosphorylation of p65, IKKα/β and IκB in the KIAA1522-downregulated cells (Fig. 5b-c, Supplementary Fig. 4C-D), whereas forced expression of KIAA1522 yielded an opposite effect, which expedited the activation of NFκB signaling (Fig. 5d, Supplementary Fig. 4E). Moreover, we found that depletion of KIAA1522 decreased the abundance of the Tumor Necrosis Factor receptors, TNFR1 and TNFR2 (Fig. 5e, Supplementary Fig. 4F-G). Overexpression of KIAA1522 significantly increased TNFR2 in parallel with the activation of p65, but not TNFR1 (Fig. 5f, Supplementary Fig. 4H). So, the up-regulation of TNFR2 may contribute to the KIAA1522-mediated TNFα-NFκB activation. The AAV-mediated deletion of KIAA1522 in *Kras*^*G12D*^-induced murine lung cancer also repressed TNFR2 and NFκB signaling, as indicated by phosphorylated IKKα/β (Fig. 5g). The results verified the regulation of TNFα-NFκB pathway in vivo. Next, we also explore the potential mechanisms underlying KIAA1522 regulating TNFR2, we found that depletion of KIAA1522 did not decrease the mRNA expression of KIAA1522 (Supplementary Fig. 5A), while KIAA1522 interacted with TNFR2 (Supplementary Fig. 5B) and contributed to protein stability of TNFR2 (Supplementary Fig. 5C).

### Correlation of KIAA1522 signature with TNFα-NFκB signaling and cisplatin responsiveness in multiple cohorts of lung adenocarcinoma patients

To further underpin the links between KIAA1522 and TNFα-NFκB signaling and cisplatin responsiveness by clinical evidences, we performed integrative analyses using transcriptome profiles of lung adenocarcinoma patients from multiple cohorts, including single cell transcriptome studies. Considering the lake of KIAA1522 probes in several platforms, we defined a KIAA1522_ POS_ REG_ GENES geneset by KIAA1522 positive regulated genes in 889 cells (Fig. 4a) to calculate KIAA1522 signature score representing KIAA1522 expression amount, in bulk- or single cell-level transcriptome assays.

**Fig. 6:**
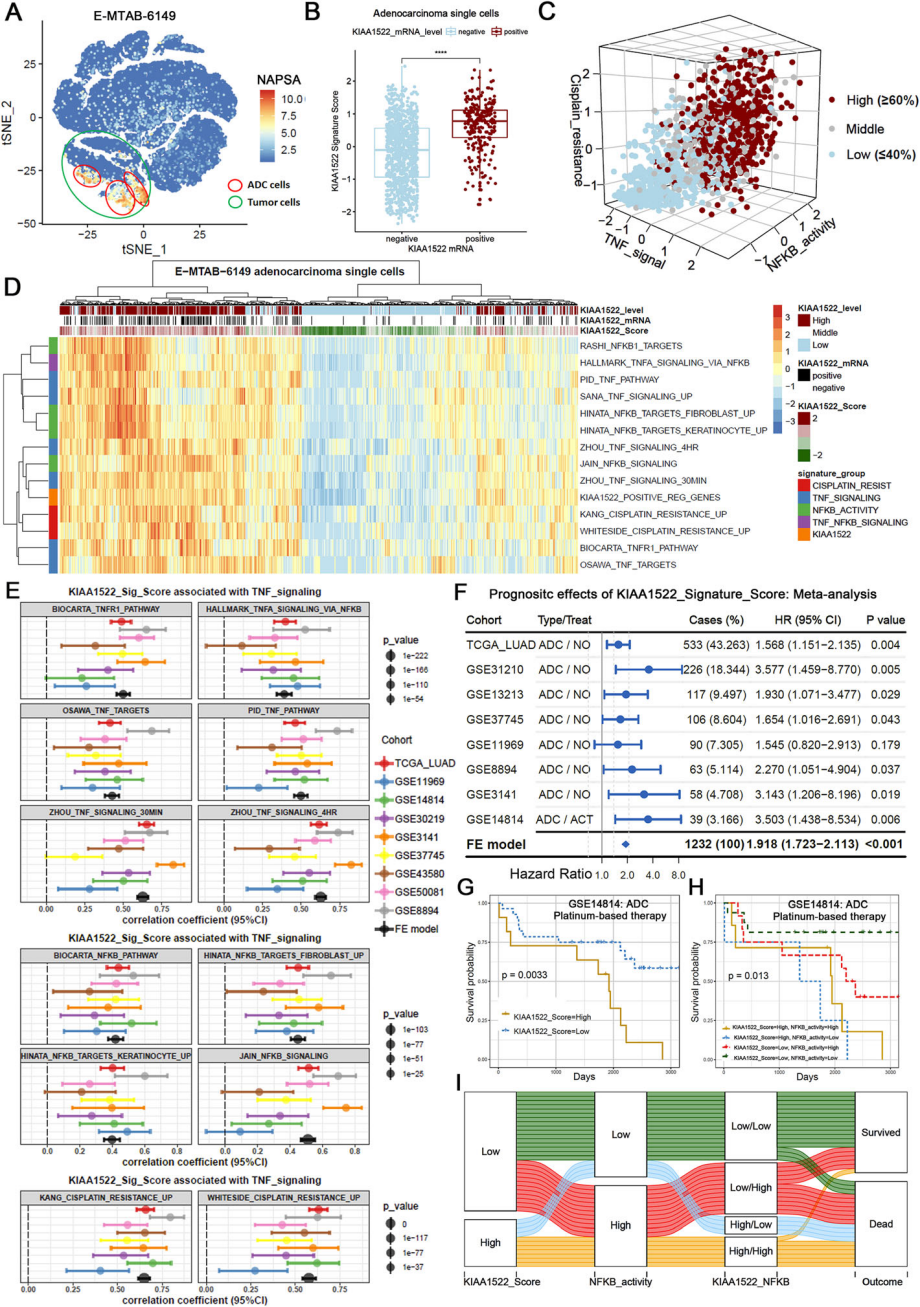
KIAA1522 signature correlates with TNF-NFκB-Cisplatin resistance in independent cohorts of adenocarcinomas. The KIAA1522 signature was generated by the collection of KIAA1522 down-stream genes and transformed to KIAA1522 signature score through GSVA algorithm. **a** Single-cell sequencing data from E-MTAB-6149 dataset was subjected to tSNE dimension reduction and clustering the NAPSA positive clusters were shown. **b** The KIAA1522 signature scores in KIAA1522 mRNA positive and negative adenocarcinoma single cells were compared, t-test, ****P < 0.0001. **c** 3D plot shows the distributions of KIAA1522 score levels according to TNFα signaling score, NFκB activity score and cisplatin resistance score. **d** Heatmap shows the scaled GSVA score of a collection of TNF-NFκB-Cisplatin resistance signatures in adenocarcinoma single-cells within E-MTAB-6149 dataset in parallel with KIAA1522 mRNA expression and KIAA1522 signature score. **e** In multi-central ADC samples, meta-analytical evaluation of the correlation between KIAA1522 signature score with TNF signaling, NFκB activity and cisplatin resistance signatures. Forest plots show the correlations together with the 95% confidence intervals, and the fixed-model estimated correlation in each geneset. **f** Systematical univariate cox analyses estimate the Hazard Ratio of KIAA1522 signature score in multiple datasets from TCGA or GEO databases. The meta-analysis was performed by fixed model to estimate the prognostic effect of KIAA1522 signature score. **g**-**h** In GSE14814 datasets, adenocarcinoma patients receiving platinum-based chemotherapy were grouped according to KIAA1522 signature score alone (**g**) or the combination of KIAA1522 signature score and NFκB activity score (**h**), then survival rates of each group were revealed by Kaplan–Meier survival analyses. **i** Alluvial diagram of KIAA1522 signature score, NFκB activity, combined KIAA1522_NFκB score and the survival outcome

In the lung cancer single cell transcriptome dataset E-MTAB-6149, we clustered the cells by Seurat software (Supplementary Fig. 6A) and filtered tumor cells by the expression of EpCAM (Supplementary Fig. 6B). Then the clusters were further classified by the expression of ADC marker NAPSA/ Napsin A and SCC marker TP63 / P63. Some groups of cells expressing NAPSA and negative for TP63 were identified as lung adenocarcinoma cells (Fig. 6a). Before in-depth analyses, we examined the reasonability of using the GSVA scores derived from KIAA1522 downstream signatures. Firstly, in the sorted adenocarcinoma cells, we observed that the KIAA1522 signature scores were much higher in the KIAA1522 mRNA positive cells than that in KIAA1522 negative cells, suggesting the consistency of KIAA1522 mRNA levels and KIAA1522 signature scores (Fig. 6b). Secondly, we analyzed the clinical relevance of cisplatin resistance signatures and found that the two selected signatures were both correlated with poor survival in the lung adenocarcinoma patients after cisplatin-based therapy (Supplementary Fig. 6C). indicating these GSVA scores recapitulated physically resistance to cisplatin. Pearson correlation analyses indicated that the KIAA1522 signature scores were correlated with the TNFα signaling, NFκB activity and cisplatin resistance associated signatures (Supplementary Fig. 6D). The 3D plot clearly exhibited that the single cells with high levels of KIAA1522 signature scores were hyperactivated in TNFα-NFκB signaling and highly resistant to cisplatin simultaneously (Fig. 6c). Besides the representative genesets, we also showed that the KIAA1522 scores were highly consistent with the GSVA scores of a handfuls of TNFα-NFκB associated signatures (Fig. 6d).

In the bulk RNA sequencing dataset TCGA-LUAD, we also observed a similar distribution of the scores of all the signatures tested including the signatures related to TNFα-NFκB signaling, cisplatin resistance and KIAA1522 signature scores (Supplementary Fig. 7A). To strengthen this result, systematic correlation analyses showed that the positive correlations were universal in multi-central datasets (Fig. 6e). The correlations of KIAA1522 signature scores with TNFα-NFκB- cisplatin resistance signatures were independent of oncogenic KRAS and TP53 mutations (Supplementary Fig. 5B). Furthermore, meta-analysis was performed to review the prognostic effects of KIAA1522 signature score in independent datasets with survival information. The results showed that high level of KIAA1522 signature score predicted poor outcome in most of the datasets (Fig. 6f). The fixed effects model estimated hazard ratio was 1.918 (1.723–2.113) with a *P*-value< 0.001, this is in agreement with the conclusion detected in our cohorts of patients. More importantly, there are 39 cases in the dataset GSE14814 received platinum-based adjuvant chemotherapy. In this specific cohort, lower level of KIAA1522 scores also implicated good therapeutic results (Fig. 6g). When considering the combined effects of KIAA1522 score and NFκB status, the result showed that the groups of patients with both low levels of KIAA1522 and NFκB activity had the best outcomes (Fig. 6h-i).

### Inhibition of NFκB signaling restores sensitivity to cisplatin in KIAA1522 overexpressed cells

**Fig. 7:**
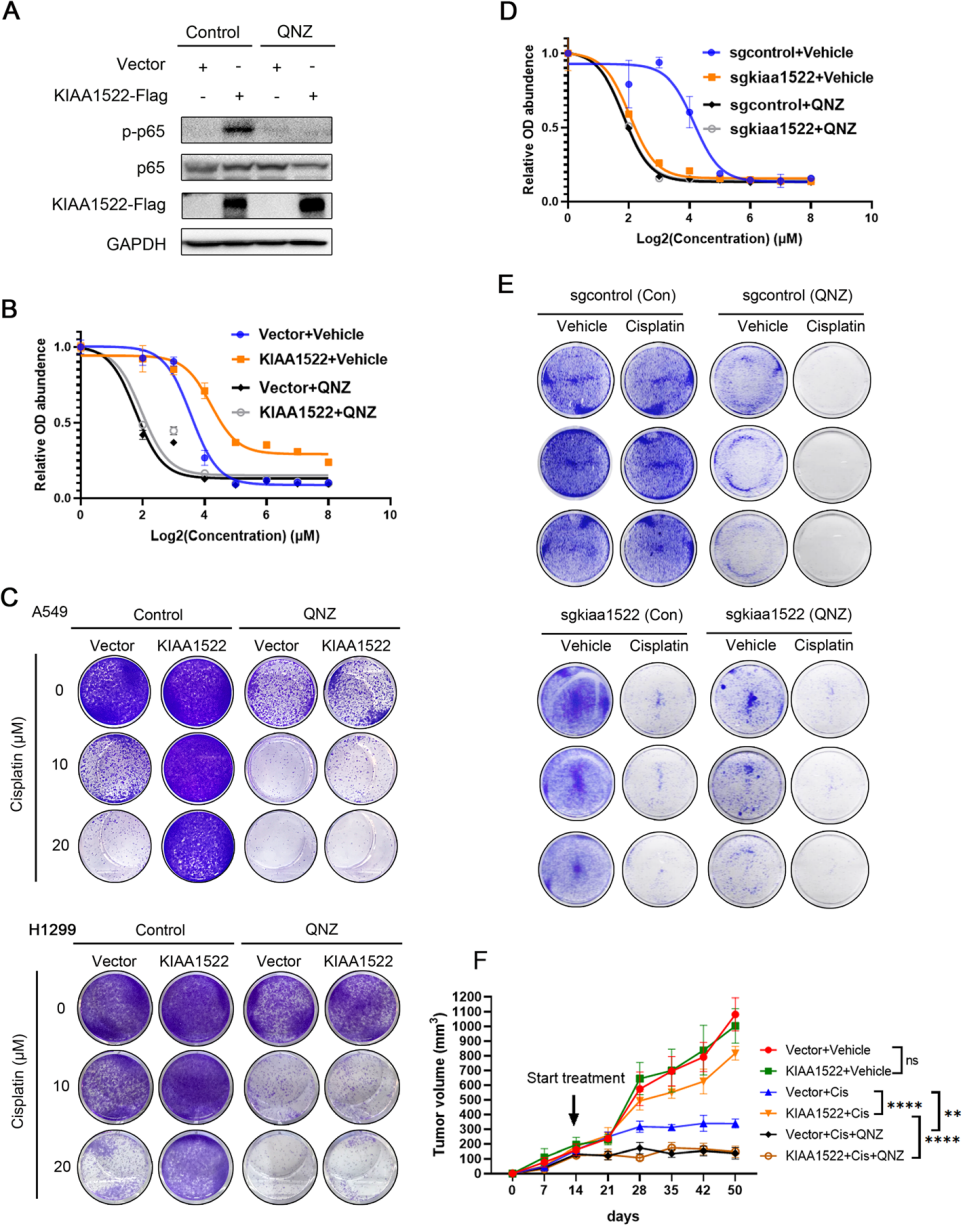
Inhibition of NFκB signaling counteracts cisplatin insensitivity in KIAA1522 hyperactivated cells. **a** Inhibitory effect of QNZ on NFκB signaling in KIAA1522-overexpressed A549 cells was tested by immunoblotting assay. **b** The control and KIAA1522-overexpressed A549 cells were treated by indicated concentration of cisplatin with or without QNZ (500 nM). Cell viability were analyzed by CCK8 assays. **c** Clonal formation assays measuring the effect of QNZ on cisplatin-induced growth inhibition in the control and KIAA1522-overexpressed A549 and H1299 cells. The cells were treated with 500 nM QNZ and the indicated concentration of cisplatin for 48 h before crystal violet staining. **d** The control and KIAA1522 depleted 889 cells were treated by different concentrations of cisplatin with or without QNZ, then analyzed by CCK8 assays to determine cell viability. **e** Clonal formation assays of the control-sgRNA/Cas9 and kiaa1522-sgRNA/Cas9 expressing 889 cells treated by QNZ and cisplatin for 48 h. **f** the control and KIAA1522-overexpressed A549 cells were subcutaneously injected into nude mice. From 14 days after cell injection to the 50th day, the mice were treated by cisplatin (7 mg/kg B.W.) once a week and QNZ (1 mg/kg B.W.) twice a week via intraperitoneal injection. Tumor volumes in the last day were compared using t-test, **P < 0.01, ****P < 0.0001

To test whether hyperactivation of NFκB signaling is critical for the KIAA1522-induced chemoresistance, we used a NFκB inhibitor QNZ to block NFκB signaling activity (Fig. 7a). QNZ ameliorated the irresponsiveness of the exogenous KIAA1522 expressed cells (Fig. 7b-c). Coherently, in the presence of QNZ, either the control cells or the KIAA1522-depleted cells yielded similar effects in response to cisplatin (Fig. 7d-e). These results substantiated that NFκB signaling is engaged in KIAA1522 mediated cisplatin resistance, and enlightened us to cooperatively use of NFκB inhibitor and cisplatin to circumvent the treatment-refractory nature of lung adenocarcinomas expressed high level KIAA1522. To exam this speculation, we performed the in vivo pharmacology experiment. The results revealed that although forced expression of KIAA1522 did not accelerate tumorigenesis, but it desensitized inhibitory effects of cisplatin in vivo. The KIAA1522-induced cisplatin resistance, like the in vitro observations, dampened by NFκB inhibition (Fig. 7f). Collectively, synergic usage of NFκB inhibitor and cisplatin counteracted the cisplatin-refractory phenotype of KIAA1522 overexpressed lung adenocarcinoma cells.

## Discussion

Nowadays, platinum-based adjuvant chemotherapy is still an irreplaceable way in routine practice to treat NSCLC patients after curative resection. Nevertheless, the efficiency is quite limited. One ideal route to optimize the usage of platinum reagents relies on customizing the patients based on histological diagnosis and the expression of tumor biomarkers [35]. Histological subtypes of non-small cell lung cancer (ADC or SCC) determine the cisplatin responsiveness [12], choice of treatments [36], or the dependency of biomarkers [37]. But effective indicators for each type of NSCLC are urgently needed. In our cohort of NSCLC patients, it is similar in the survival rate of ADC and SCC in either chemo-naive or chemotherapy-receiving patients (Supplementary Fig. 1E). Although the treatment by platinum-based chemotherapy slightly alleviated tumoral death, but failed to reach the statistical threshold (Supplementary Fig. 1F). In this background, it is meaningful to find that the expression of KIAA1522 characterizes a hypersensitive group in the lung adenocarcinoma, emphasizing KIAA1522 a potential indicator to forecast therapeutic consequence of platinum-based chemotherapy. Moreover, we found that in the GSE14814 dataset [38], containing expression profiles of ADC patients receiving cisplatin- doublet chemotherapy, KIAA1522 acts in a similar degree to predict therapeutic efficiency, supporting the robustness of this predictor.

Previously, we have reported that the expression of KIAA1522 was altered even in early lung tumor cells in bronchial brushing specimens [21]. It can be readily detected using commercial antibodies. The clear immunohistochemical staining signal enables KIAA1522 suitable for pathological diagnosis in the clinical application, that is the prerequisite in selecting candidate proteins for our clinical analyses. In contrast to the strong staining signal in tumoral tissues, the expression of KIAA1522 were extremely low in nearly 500 non-tumoral lung tissues in our cohort, as well as in the TCGA datasets. We have also identified KIAA1522 as a prognostic factor for NSCLC in a former study [22]. Here we elucidate the predictive role of KIAA1522 detailly with the verification in multicentered independent cohorts. Unlike the controversial results from squamous cell carcinoma, the prognostic values of KIAA1522 in lung adenocarcinoma were highly consistent in almost all studied datasets from several databases. The convincing clinical results implied the pleiotropic roles of this gene participating in variant steps of lung adenocarcinoma, and encourage us conducted the functional studies about KIAA1522. To do this robustly, we employed a *Kras*^*G12D*^-induced murine lung adenocarcinoma model coupled by Cas9/sgRNA-mediated gene editing [39]. This in vivo system easily generated lung cancer cell specific-knockout mice, empowering the genetical evaluation of KIAA1522 function in an economic effective manner. The in vivo experiments using genetic mice model represent the clinical results, reinforcing the requirement of KIAA1522 to both tumor development and intrinsic resistance to cisplatin.

Despite the extraordinary biological effects of KIAA1522 in functional assays in vitro and in vivo, the molecular details of this protein remain elusive. KIAA1522 is neither an enzyme or a receptor, making it undruggable based on the existing knowledge. However, uncovering a targetable molecular event key to KIAA1522-downstream pathways may open an alternative avenue to fight against KIAA1522-regulated malignancy. To this end, we identified the activation of NFκB signaling was engaged in KIAA1522-mediated cisplatin-resistance. Notoriously recognized the significance of NFκB pathway in distinct biological processes [13], so it needs to be tightly controlled to ensure appropriate onset. As a bona fide upstream activator to stimulate NFκB signaling, TNFα binds to the TNFR1 or TNFR2 receptor complexes to invigorate NFκB signal transduction [40]. Here, we found that KIAA1522 may modulating NFκB activity via TNFR2, a TNFα receptor transmitting only anti-apoptotic signals [41], that is in concordance with the roles of KIAA1522 in lung adenocarcinomas. Further results suggested that the KIAA1522-mediated up-regulation of TNFR2 may occur in the post-transcriptional level, that is KIAA1522 interacting and stabilizing TNFR2. Since our knowledge about the molecular function of KIAA1522 protein were limited, and little is known about the turnover mechanism of TNFR2 protein. So, whether and how KIAA1522 directly work on TNFR2 to active NFκB signal was still far from clear. This is a major limitation of this work that need further exploration. Nonetheless, our pre-clinical studies prove that it is reasonable to inhibit NFκB activity to reverse the recalcitrant effects of KIAA1522 overexpressed cancer cells. On the other hand, the findings shed light on novel NFκB regulatory mechanism and clinical implication.

Collectively, this work proposes a rational strategy to characterize and treat lung adenocarcinomas. The protein levels of KIAA1522 should be firstly detected as a biomarker to stratify the patients into cisplatin sensitive and insensitive groups. The patients expressed low level of KIAA1522 may be prone to benefit from conventional platinum-based adjuvant chemotherapy. In the opposite, the lung adenocarcinomas with high KIAA1522 expression may be more willing to escape from cisplatin induced regression, which should be treated synergistically by NFκB inhibitor and platinum-based reagents to restore chemosensitivity. This methodology may help to magnify the therapeutic efficiency of platinum-based chemotherapy.

## Conclusions

We found that KIAA1522 acts as an indicator of poor outcome of platinum-based therapy in lung adenocarcinomas. KIAA1522 potentiates the TNFα-NFκB signaling which leads to cisplatin resistance. Our findings suggest that combined use of NFκB inhibitor and platinum-contained compounds may be active against KIAA1522 overexpressed lung adenocarcinomas.

## Supplementary information

**Additional file 1: Supplementary table and figures. Table S1.** Basic clinicopathologic data of tissue samples from patients with NSCLC. **Supplementary Fig. 1.** Multivariate cox analysis of KIAA1522 protein levels in NSCLC patients. **Supplementary Fig. 2.** KIAA1522 expression is elevated and positively correlated with poor prognosis in TCGA NSCLC datasets. **Supplementary Fig. 3.** KIAA1522 regulates TNF-NFκB downstream genes. **Supplementary Fig. 4.** KIAA1522 enhances the activation of TNFα-NFκB signaling (related to Figure 5). **Supplementary Fig. 5.** KIAA1522 interacts and stabilizes TNFR2.** Supplementary Fig. 6.** Signature correlation analysis in single-cell RNA sequencing data. **Supplementary Fig. 7.** Correlation of KIAA1522 signature score with TNF-NFκB and cisplatin resistance signatures.

## Data Availability

The datasets generated and/or analyzed during the current study are available in the Gene Expression Omnibus repository (GEO; https://www.ncbi.nlm.nih.gov/geo/) through GEO accessions: GSE146072, GSE3141, GSE8894, GSE13213, GSE11969, GSE37745, GSE31210, GSE30219, GSE50081, GSE43580 and GSE14814. The TCGA datasets are available in the GDC portal (https://portal.gdc.cancer.gov/).

## Abbreviations

NSCLC: Non-small cell lung cancer
ADC: Lung adenocarcinomas
SCC: Squamous cell carcinomas (SCC)
GSEA: Gene set enrichment analysis
GSVA: Gene set variation analysis analysis
AAV: Adeno-associated virus
