# Supplementary material for: KIAA1522 potentiates TNFα-NFκB signaling to antagonize platinum-based chemotherapy in lung adenocarcinoma
Source: J Exp Clin Cancer Res. 2020 Aug 27;39:170. doi: 10.1186/s13046-020-01684-x (PMC7450600; doi:10.1186/s13046-020-01684-x)
Supplement: Supplementary file 1 — Additional file 1: Supplementary table and figures. Table S1. Basic clinicopathologic data of tissue samples from patients with NSCLC. Supplementary Fig. 1. Multivariate cox analysis of KIAA1522 protein levels in NSCLC patients. Supplementary Fig. 2. KIAA1522 expression is elevated and positively correlated with poor prognosis in TCGA NSCLC datasets. Supplementary Fig. 3. KIAA1522 regulates TNF-NFκB downstream genes. Supplementary Fig. 4. KIAA1522 enhances the activation of TNFα-NFκB signaling (related to Figure 5). Supplementary Fig. 5. KIAA1522 interacts and stabilizes TNFR2. Supplementary Fig. 6. Signature correlation analysis in single-cell RNA sequencing data. Supplementary Fig. 7. Correlation of KIAA1522 signature score with TNF-NFκB and cisplatin resistance signatures. [file 13046_2020_1684_MOESM1_ESM.docx]

**Supplementary table**

**Table S1. Basic clinicopathologic data of tissue samples from patients with NSCLC**

| **Parameter** | **No. of tissue samples** |
| --- | --- |
| Age |  |
| ＞60 | 319 |
| ≤60 | 264 |
| NA | 15 |
| Sex |  |
| Male | 434 |
| Female | 149 |
| NA | 15 |
| Tumor type |  |
| SCC | 314 |
| ADC | 269 |
| NA | 15 |
| Tumor stage^a^ |  |
| 0 | 1 |
| I | 187 |
| II | 222 |
| III+IV | 173 |
| NA | 15 |
| T status |  |
| Tis | 1 |
| T1 | 18 |
| T2 | 373 |
| T3 | 96 |
| T4 | 40 |
| NA | 70 |
| N status |  |
| N0 | 304 |
| N1-3 | 279 |
| NA | 15 |
| M status |  |
| M0 | 572 |
| M1 | 11 |
| NA | 15 |
| Tumor differentiation^b^ |  |
| Well-Moderate | 300 |
| Poorly | 282 |
| NA | 16 |
| Treatment |  |
| No treatment | 319 |
| Chemotherapy | 132  0 |
| Radiotherapy | 11 |
| Radiochemotherapy | 29 |
| NA | 107 |
|  |  |

^a^ Tumor stage was classified according to the 7th edition of the International Union against Cancer (UICC) Tumor Node Metastasis (TNM) classification of malignant tumors.

^b^ Tumor differentiation was based on the criteria of the 2004 World Health Organization Classification of Tumors.

**Supplementary Figures**

**
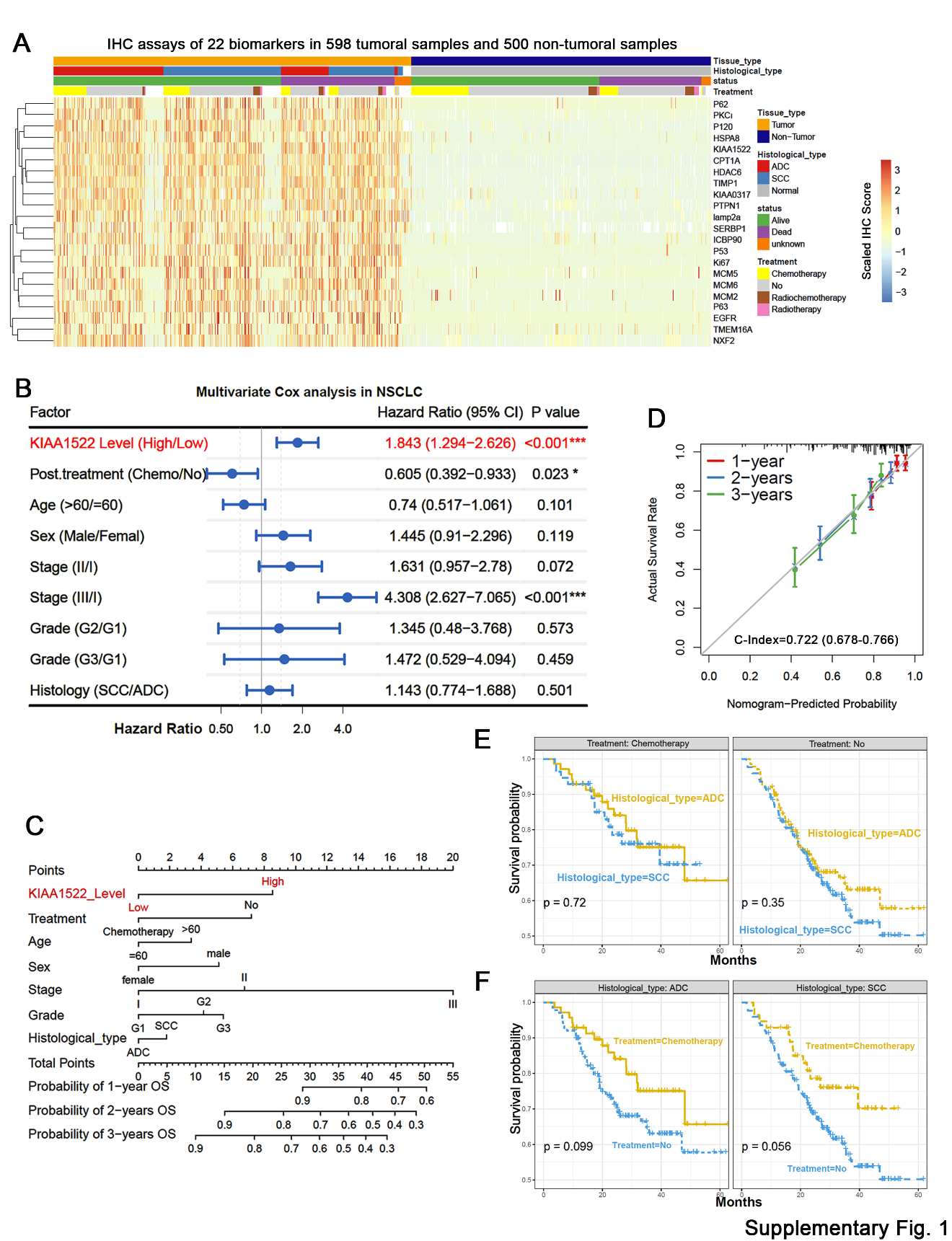
**

**Supplementary Fig. 1. Multivariate cox analysis of KIAA1522 protein levels in NSCLC patients**

(A) Screening of proteins that were elevated in tumoral tissues in comparison with the non-tumoral adjacent lung tissues. Heatmap shows the scaled IHC scores of 22 tested proteins in a total of 1098 tissue samples from 598 NSCLC patients, including 598 tumoral tissues and 500 non-tumoral tissues. There are missing values shown by white boxes. (B) Multivariate cox analyses to estimate the hazard ratio of KIAA1522 levels, age, sex, stage, grade and histological types in the NSCLC patients with or without post-surgical chemotherapy. (C) A Nomogram model involving KIAA1522 expression levels to predict the one-, two-, and three-years overall survival rates of NSCLC patients. (D) The comparison between the Nomogram predicted survival probability and the actual survival rate. (E) Kaplan–Meier curves compare the overall survival between different histological types in chemo-naive or treatment-experienced NSCLC patients. (F) Kaplan–Meier curves compare the overall survival between chemo-naive and treatment-experienced patients in ADC or SCC groups respectively.

**
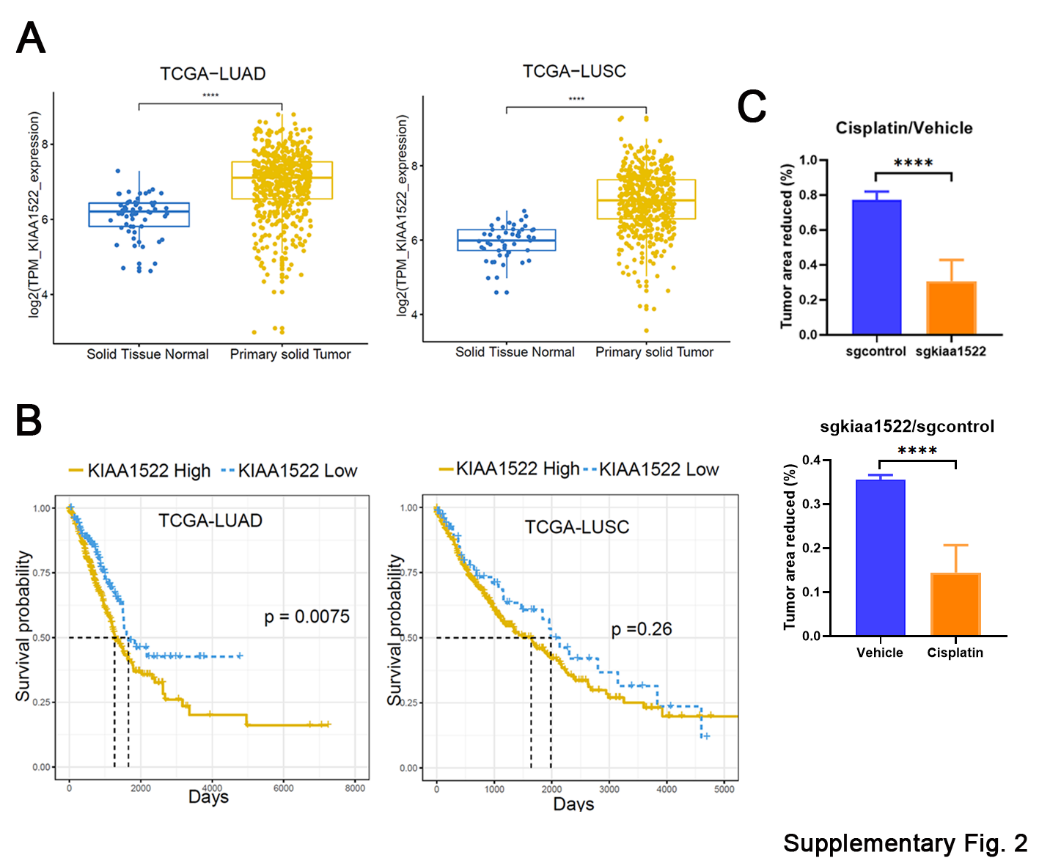
**

**Supplementary Fig. 2. KIAA1522 expression is elevated and positively correlated with poor prognosis in TCGA NSCLC datasets**

(A) The mRNA levels of KIAA1522 in non-tumoral and tumor tissues in TCGA-LUAD and TCGA-LUSC datasets. (B) Kaplan–Meier curves show the association of KIAA1522 expression and overall survival in TCGA-LUAD and TCGA-LUSC datasets. (C) The statistics of percentage of tumor area in the lung between different groups. Lung tumor burden reduced by cisplatin treatment in control-sgRNA- and kiaa1522-sgRNA treated mice, and tumor burden decreased by kiaa1522 deletion with or without cisplatin management were compared respectively. t-test, *****P*<0.0001.


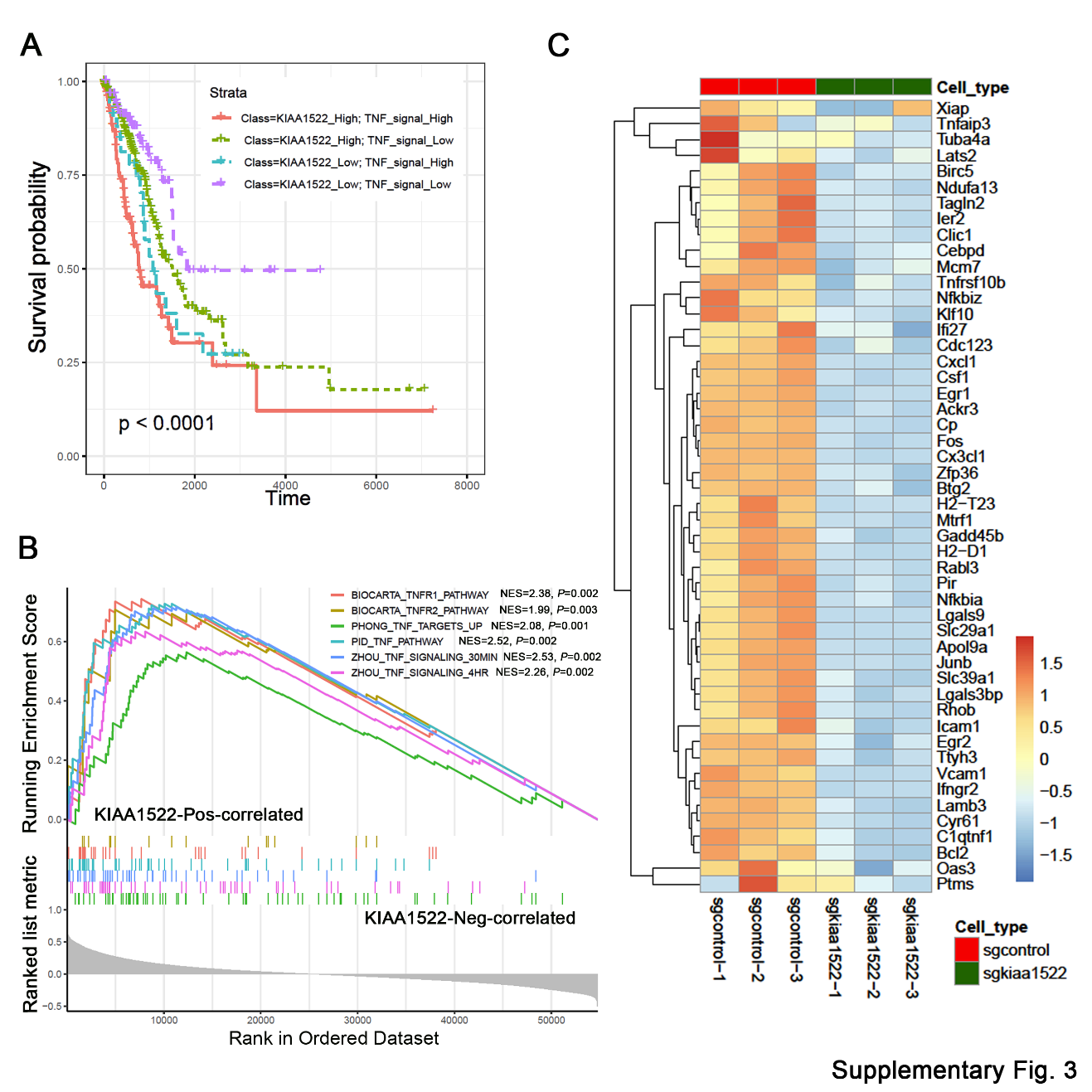


**Supplementary Fig. 3. KIAA1522 regulates TNF-NFκB downstream genes**

(A) The patients in TCGA-LUAD cohort were classified by both KIAA1522 expression and TNFα signal levels activity into four groups and subjected to Kaplan–Meier overall survival analysis. The activity of NFκB signaling was determined by the GSVA score of the geneset ZHOU_TNF_SIGNALING_30MIN. (B) The genes were ranked by the Pearson correlation coefficient with KIAA1522 expression in TCGA-LUAD datasets. Then, the GSEA assays were performed using a set of TNFα signaling related genesets to evaluate the correlation of KIAA1522 expression level with TNFα signaling. (C) The heatmap shows the scaled FPKM-values of a set of TNF-NFκB related genes in control or kiaa1522 sgRNA expressed 889 cells.

**
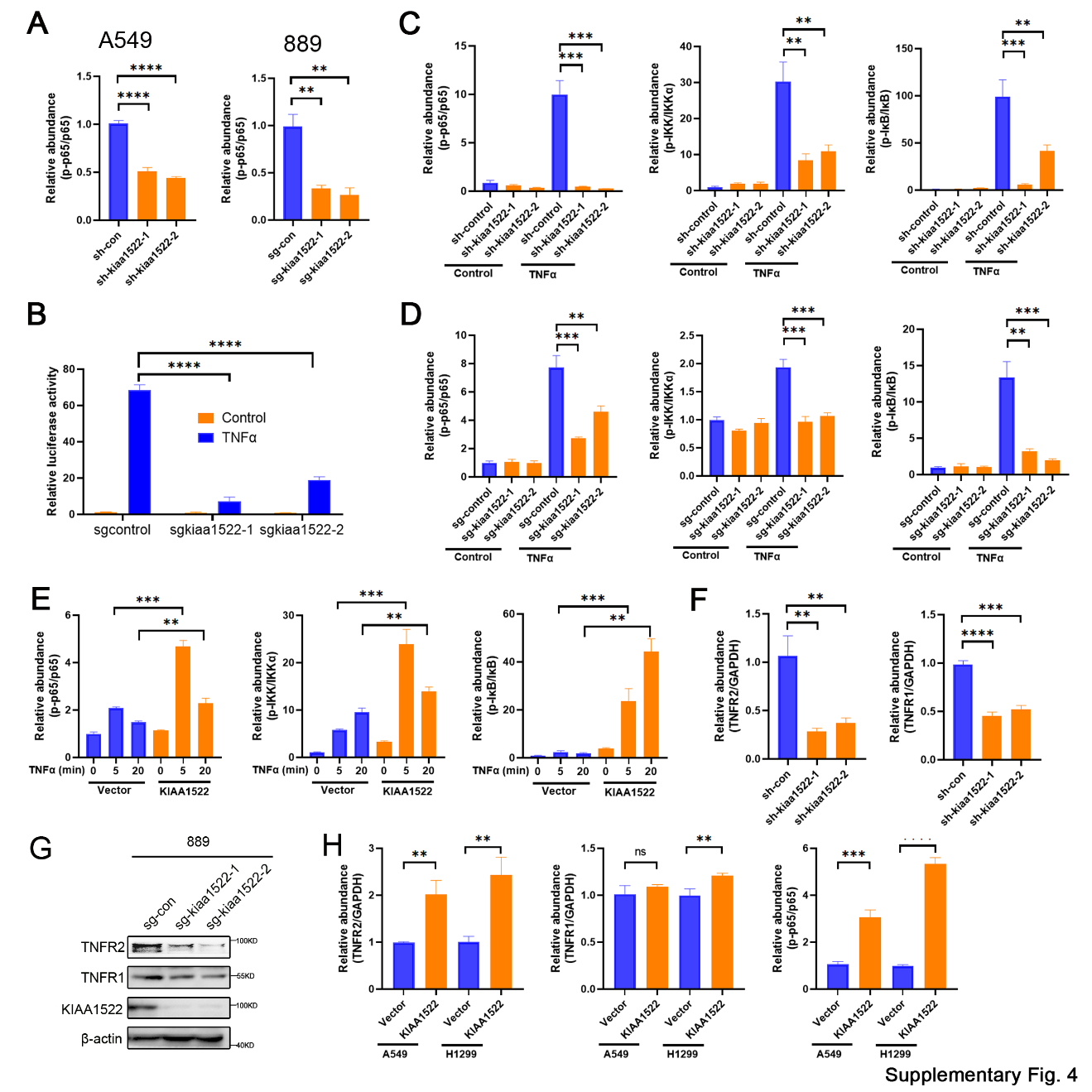
**

**Supplementary Fig. 4. KIAA1522 enhances the activation of TNFα-NFκB signaling (related to Figure 5)**

(A) Quantitative and statistical analyses of western blotting assays in Fig. 5A. (B) Luciferase reporter assays were performed to examine the activity of NFκB signaling pathway in control and KIAA1522-depleted 889 cells treated with 10ng/ml TNFα for 5 minutes. t-test, *****P*<0.001. (C-F) Quantitative and statistical analyses of western blotting assays in Fig. 5B (C), Fig. 5C (D), Fig. 5D (E) and Fig. 5E (F). (G) Western blotting assays show the expression of TNFR2 and TNFR1 in KIAA1522 depleted 889 cells. (H) Quantitative and statistical analyses of western blotting assays in Fig. 5F. Data are mean ± SD, ** *P*<0.01, *** *P*<0.001, **** *P*<0.0001, tested by t-test, n=3.

**
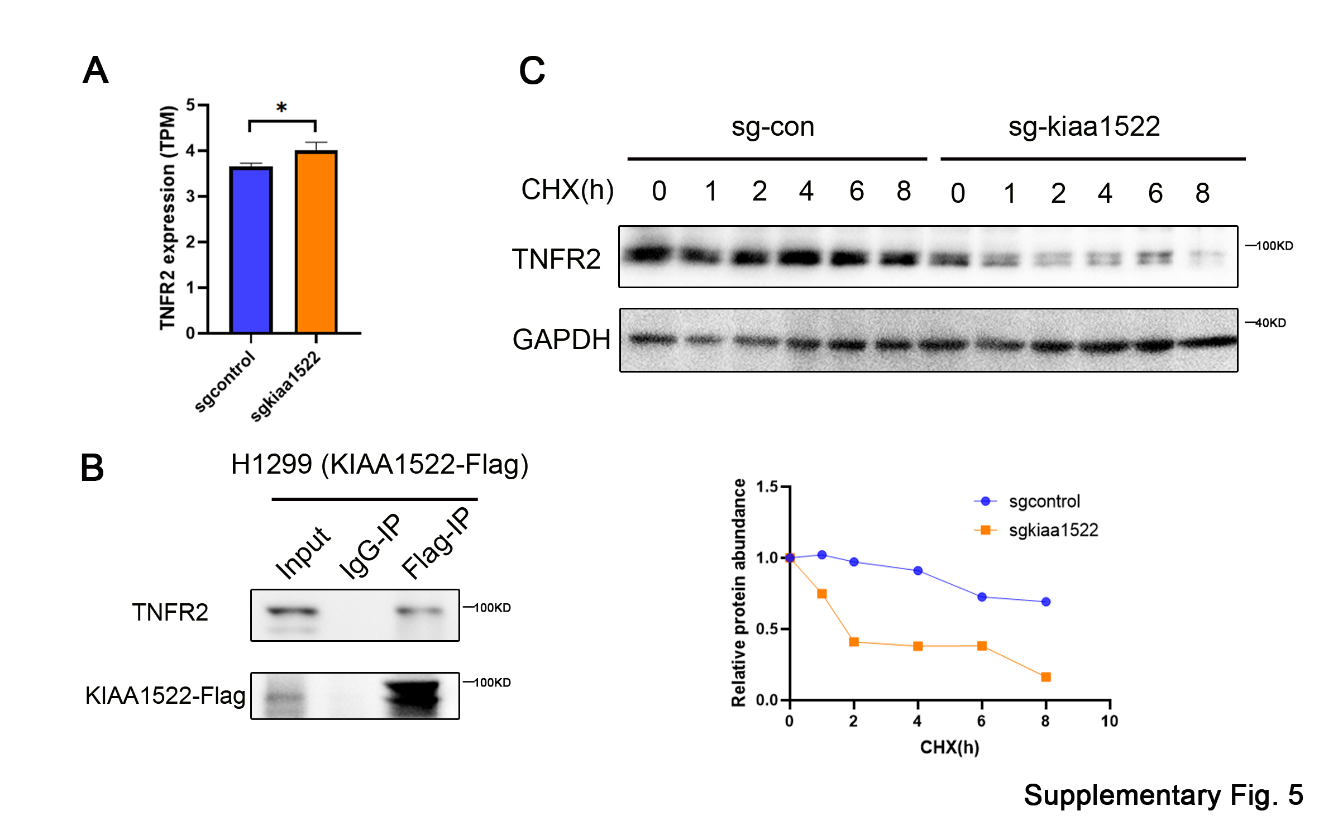
**

**Supplementary Fig. 5. KIAA1522 interacts and stabilizes TNFR2**

(A) Comparing the TPM values of TNFR2 in the control and sgkiaa1522/Cas9 expressing 889 cells from RNA-seq data. (B) The cell lysates of KIAA1522-Flag expressing H1299 cells were immunoprecipitated by anti-Flag antibody and subjected to western blot assay to detect the co-immunoprecipitated TNFR2. (C) The control and sgkiaa1522/Cas9 expressing 889 cells were treated by Cycloheximide (200 μg/ml) for the indicated time period. The cell lysates were analyzed by immunoblotting.

**
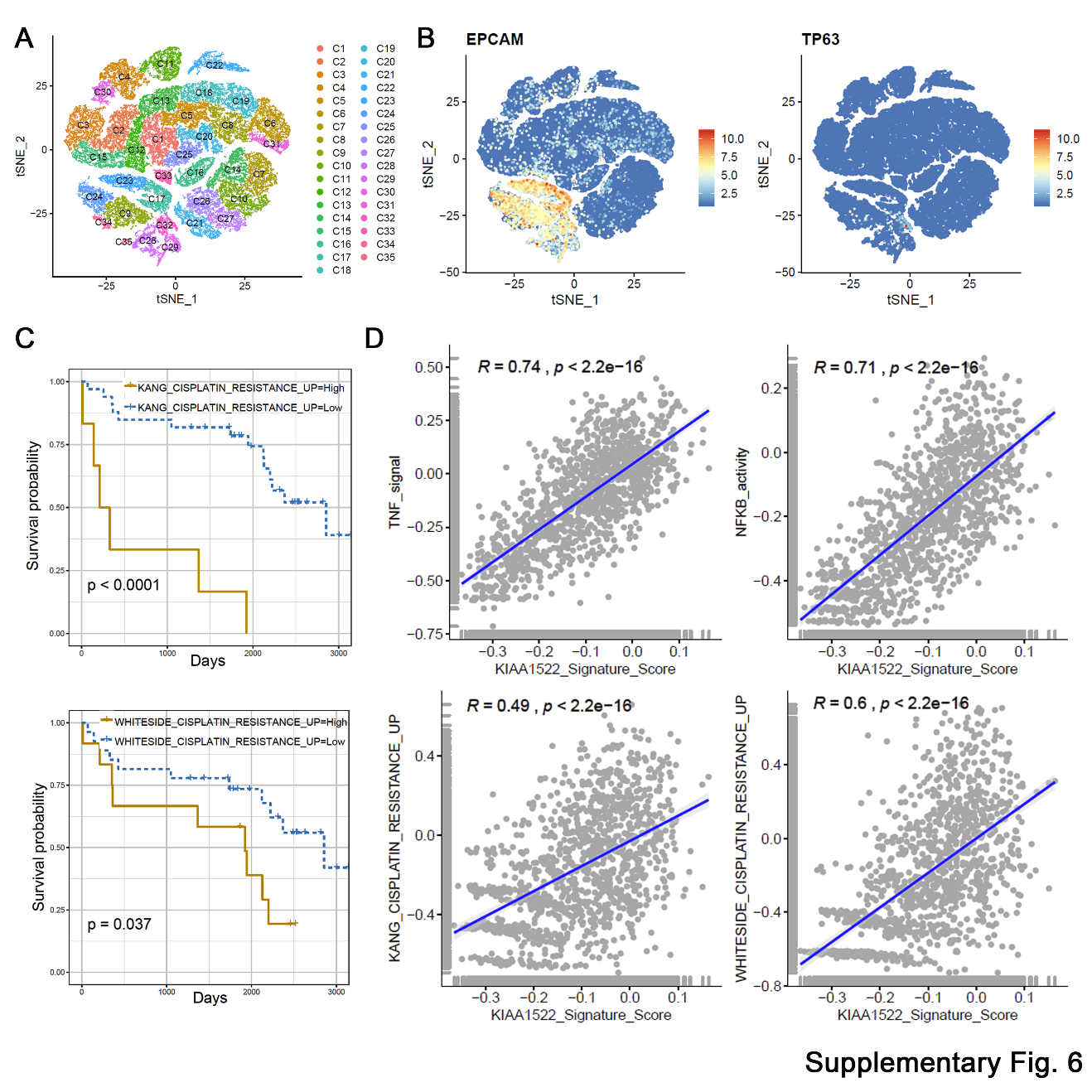
**

**Supplementary Fig. 6. Signature correlation analysis in single-cell RNA sequencing data**

(A) The tSNE plot shows the clusters of single cells in E-MTAB-6149 dataset. (B) The expression of EPCAM and TP63 in single cells in E-MTAB-6149 dataset. (C) Two cisplatin resistance signatures in MSigDB, KANG_ CISPLATIN_ RESISTANCE_ UP and WHITESIDE_ CISPLATIN_ RESISTANCE_ UP were demonstrated to be positively associated with poor survival in the adenocarcinoma patients receiving adjuvant chemotherapy in GSE14814 datasets. (D) Scatterplot show the correlation of KIAA1522 signature score with TNF-NFκB-Cisplatin resistance signatures in adenocarcinoma cells within E-MTAB-6149 dataset.

**
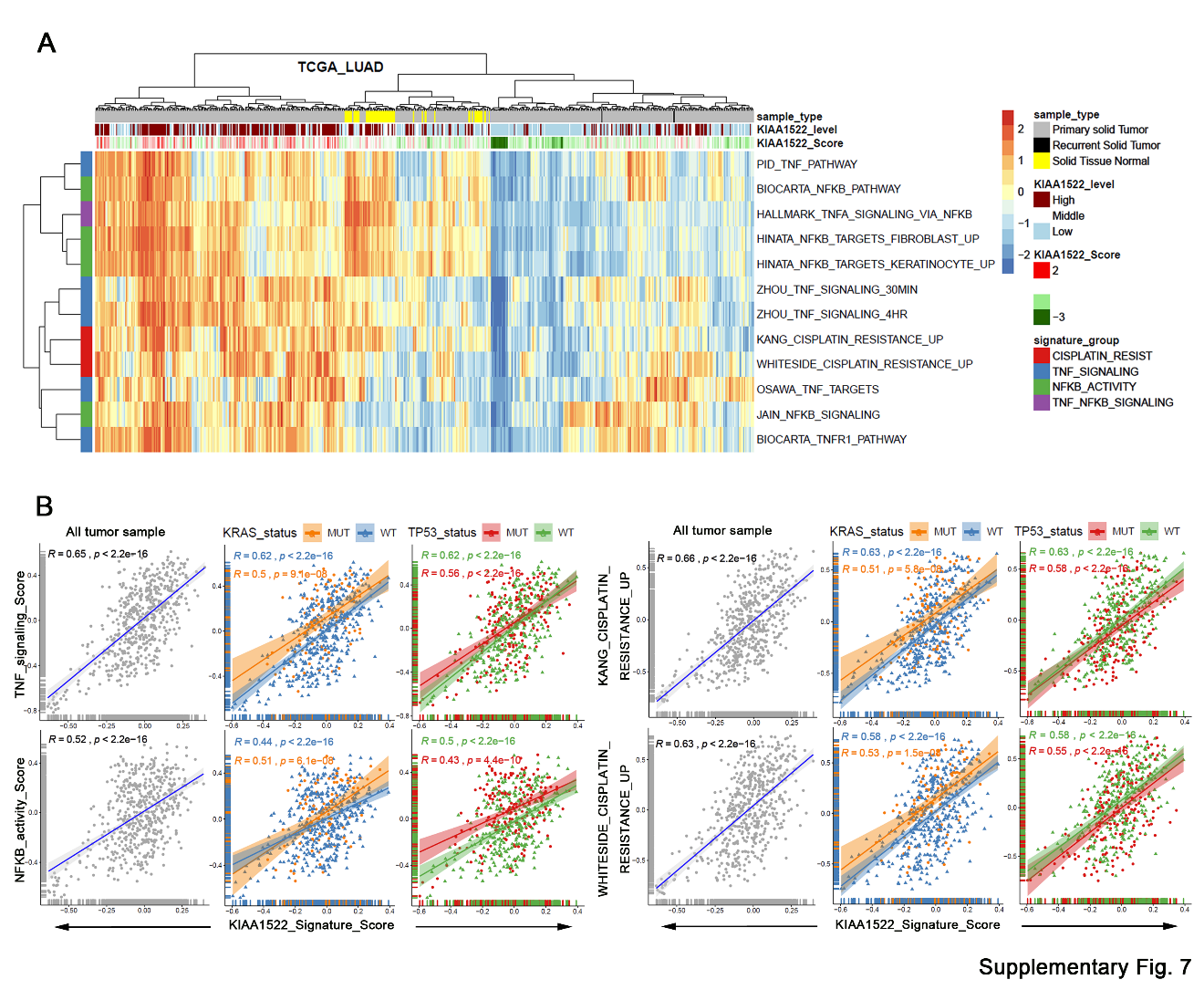
**

**Supplementary Fig. 7. Correlation of KIAA1522 signature score with TNF-NFκB and cisplatin resistance signatures**

(A) Heatmap shows the scaled GSVA score of the indicated TNF- NFκB-Cisplatin resistance signatures in each sample within TCGA-LUAD datasets in parallel with KIAA1522 signature score. (B) Scatterplots show the correlation between GSVA-derived KIAA1522 signature score and TNF-NFκB and cisplatin resistance signatures in TCGA-LUAD datasets. The Pearson correlation assays were conducted in all tumor samples, KRAS mutation, and TP53 mutation samples, respectively.
